# Integrating Siderophore Substructures in Thiol-Based Metallo-β-Lactamase Inhibitors

**DOI:** 10.3390/molecules28041984

**Published:** 2023-02-20

**Authors:** Marco J. Rotter, Sabrina Zentgraf, Lilia Weizel, Denia Frank, Luisa D. Burgers, Steffen Brunst, Robert Fürst, Anna Proschak, Thomas A. Wichelhaus, Ewgenij Proschak

**Affiliations:** 1 Institute of Pharmaceutical Chemistry, Goethe University Frankfurt, Max-von-Laue-Straße 9, D-60438 Frankfurt, Germany; 2 Institute of Medical Microbiology and Infection Control, University Hospital Frankfurt, Paul-Ehrlich-Straße 40, D-60596 Frankfurt, Germany; 3 Institute of Pharmaceutical Biology, Goethe University Frankfurt, Max-von-Laue-Straße 9, D-60438 Frankfurt, Germany

**Keywords:** metallo beta lactamases, inhibitors, siderophore, sideromycin, antimicrobial resistance

## Abstract

Metallo beta lactamases (MBLs) are among the most problematic resistance mechanisms of multidrug-resistant Gram-negative pathogens due to their broad substrate spectrum and lack of approved inhibitors. In this study, we propose the integration of catechol substructures into the design of thiol-based MBL inhibitors, aiming at mimicking bacterial siderophores for the active uptake by the iron acquisition system of bacteria. We synthesised two catechol-containing MBL inhibitors, as well as their dimethoxy counterparts, and tested them for in vitro inhibitory activity against NDM-1, VIM-1, and IMP-7. We demonstrated that the most potent catechol-containing MBL inhibitor is able to bind Fe^3+^ ions. Finally, we could show that this compound restores the antibiotic activity of imipenem in NDM-1-expressing *K. pneumoniae*, while leaving HUVEC cells completely unaffected. Thus, siderophore-containing MBL inhibitors might be a valuable strategy to overcome bacterial MBL-mediated resistance to beta lactam antibiotics.

## 1. Introduction

**Figure 1:**
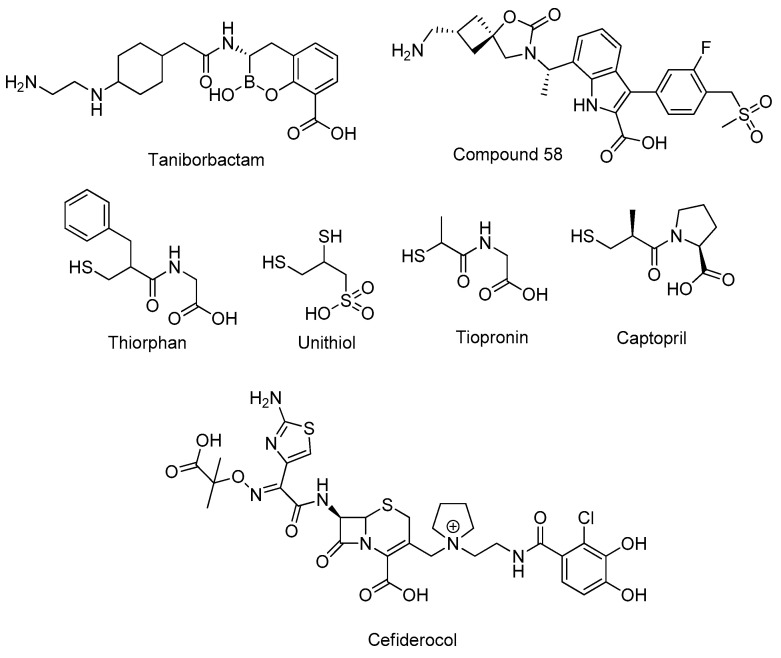
Upper line: most advanced MBL inhibitors. Middle line: approved thiol-containing drugs shown to inhibit MBLs. Bottom line: Approved sideromycin cifederocol.

Multidrug-resistant Gram-negative pathogens are regarded as one of the major threats for human health. In the fight against these pathogens, approved anti-infectives are becoming increasingly ineffective. β-Lactamases, which inactivate β-lactam antibiotics, are produced by many multi-resistant Gram-negative bacteria and represent the most effective resistance mechanisms. Therefore, the inhibitors of β-lactamases are considered as effective antibiotic adjuvants, which can restore the activity of β-lactam antibiotics [1]. β-Lactamases can be separated in accordance to the underlying hydrolysis mechanism. Serine-β-lactamases (SBLs) use a nucleophilic serine to cleave the β-lactam core, while metallo-β-lactamases (MBLs) enable the nucleophilic attack of an activated water molecule, coordinated by one or two Zn^2+^ ions in the active site [2]. While a number of SBL inhibitors, in combination with ß-lactam antibiotics, are approved for therapy, no inhibitor of MBLs has yet been approved. The most advanced MBL inhibitor, taniborbactam (Figure 1), is currently under investigation in clinical phase III trials for the treatment of complicated urinary tract infections [3].

Taniborbactam exhibits a cyclic boronate moiety that mimics the transition state of the β-lactam hydrolysis and coordinates to Zn^2+^ ions in the active site of MBLs [4]. Furthermore, Brem et al. recently reported that indole-2-caboxylate-based compound 58 is a β-lactam mimetic, which displays excellent in vitro and in vivo adjuvant activity in combination with meropenem [5]. Another attractive option to inhibit MBL is offered by thiol-containing compounds [6,7]. The thiol moiety is a soft Lewis base which tightly coordinates to Zn^2+^ ions; thereby, it displaces the activated water molecule and inhibits the MBL. Different thiol-based MBL inhibitors have been reported so far, including the approved drugs captopril, tiopronin, thiorphan [8], and unithiol [9] (Figure 1). Numerous thiol-containing inhibitors potently inhibit MBLs in vitro, however, to our best knowledge, none of these promising compounds have reached clinical trials as antibiotic adjuvance so far.

One of the major drawbacks of the previously reported thiol-containing inhibitors is the comparably high lipophilicity, which is considered to be a limiting factor for the transport of the inhibitor through the outer membrane of Gram-negative pathogens into periplasm [10]. One of the strategies to overcome this drawback is the exploitation of the bacterial iron-acquisition system, also known as siderophore system. Siderophores are high-affinity iron chelators that are produced and excreted by bacteria and are actively re-imported as siderophore–iron complexes [11]. Covalent conjugates of siderophores with antibiotics are also known as sideromycins; they mimic the siderophore–iron complexes to be actively imported by bacteria [12]. Sideromycins are not only synthetic products, as naturally occurring sideromycins are produced, e.g., by Streptomyces species [13]. Various combinations of siderophores and antibiotic agents have been reported so far, ultimately leading to the recent approval of cifederocol (Figure 1). Cifederocol is a synthetic sideromycin that comprises a cephalosporin antibiotic linked to a catechol moiety, which is responsible for iron chelation. This combination makes cifederocol a highly effective antibiotic active against multi-resistant pathogens [14,15]. In this study we applied the sideromycin concept to thiol-based MBL inhibitors by integrating catechol moieties. 

## 2. Results

### 2.1. Design of Siderophore-Containing MBL Inhibitors

**Figure 2:**
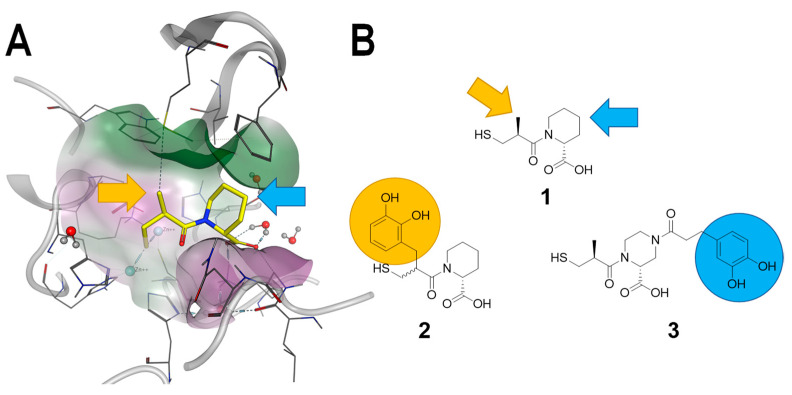
Design strategy of siderophore-containing MBL inhibitors. (**A**). X-ray structure of NDM-1 in complex with compound **1** (PDB code 6LJ0). Yellow and blue arrows indicate the positions which are sterically and chemically accessible for introduction of larger moieties. (**B**). Yellow and blue arrows indicate chemically tractable positions to introduce catechol moieties in compound **1**, which results in the design of compounds **2** and **3**.

In this study, we report the incorporation of catechol moieties into thiol-based MBL inhibitors. Ma et al. [16] and us [10] have previously reported on D-pipecolic acid-based MBL inhibitors. The X-ray structure of compound **1** ((2*R*)-1-[(2*S*)-2-methyl-3-sulfanyl-propanoyl]piperidine-2-carboxylic acid) in complex with NDM-1 has been published by Ma et al. and guided our design strategy to introduce an iron chelating moiety. Visual examination of the NDM-1 in complex with compound 1 (PDB code 6LJ0 [16]) revealed that while the thiol and the carboxylate moieties of compound **1** are tightly involved in directed interactions with the protein, the α-methyl moiety of the 3-mercapto-2-methylpropanoic acid part (yellow arrow), as well as the 4-position of the D-pipecolic acid part (blue arrow), are exposed to solvent (Figure 2A). This observation is in perfect agreement with the structure–activity relationship studies performed by Büttner et al. where they could show that substitutions in these positions are tolerated by NDM-1, VIM-1, and IMP-7 [10]. Therefore, we proposed that compounds **2** and **3**, which exhibit a catechol moiety at the methyl position, could be potential siderophore-containing MBL inhibitors (Figure 2B).

### 2.2. Synthesis of Potential Siderophore-Containing MBL Inhibitors **2** and **3**

**Scheme 1:**
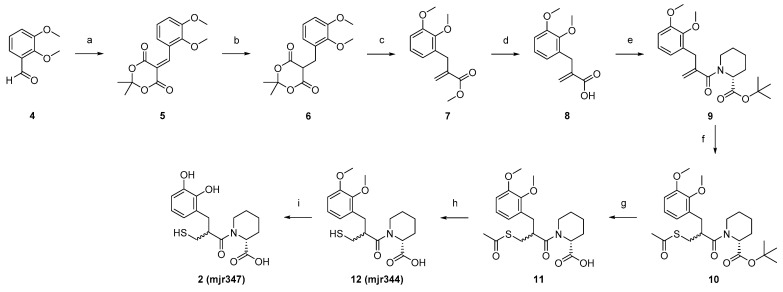
(a) Meldrum´s acid, AcOH, i-PrOH/EtOAc, 75 °C, 3 h; (b) NaBH_4_, AcOH, DCM, 0 °C–r.t., 2.25 h; (c) Eschenmoser´s salt, MeOH, 70 °C, 18 h; (d) NaOH, 100 °C, 1 h, µw; (e) 1. SOCl_2_, DCM/DMF, 50 °C, 3 h, 2. *t*-Butyl-(2R)-2-piperidinecarboxylate 19, DIPEA, DCE/DMF, 90 °C, 1 h, µw; (f) Thioacetic acid, rt, 66 h; (g) TFA, DCM, 50 °C, o.n.; (h) NH_3_ (25%), H_2_O, 0 °C–rt, 2.5 h; and (i) BBr_3_, DCM, −78 °C–r.t., o.n.

For the synthetic implementation of compounds **2** and **3,** we followed the synthetic route established by Büttner et al. [10] (Scheme 1). The preparation of compound **2** started from 2,3-dimethoxy benzaldehyde **4**, which was coupled in a Knoevenagel-like condensation with Meldrum’s acid to obtain **5**. The reduction of **5** was accomplished with sodium borohydride. A Mannich-like cycloelimination of intermediate **6** with Eschenmoser´s salt led to methyl acrylate **7**, which was subsequently converted to acrylic acid **8** via ester hydrolysis. Compound **8** was coupled with *t*Bu-protected *R*-pipecolinic acid **19** in a Schotten–Baumann reaction. The Michael addition of thioacetic acid to the double bond of **9** yielded the fully protected precursor **10**. Three deprotection steps led to the desired compound **2,** wherein the dimethoxy intermediate **12** was also characterised as a final compound for use as potential non-chelating control.

**Scheme 2:**
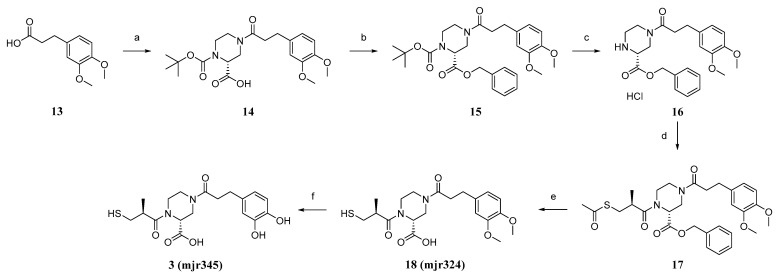
(a) 1. SOCl_2_, 90 °C, 2 h, 2. (*R*)-1-Boc-piperazine-2-carboxylic acid, DCM/pyridine/DMF, r.t., 20 h; (b) Benzyl bromide, KHCO_3_, 65 °C, 18 h; (c) HCl, DCM, r.t., o.n.; (d) 1. *D*-(-)-3-Acetylthio-2-methylpropionic acid, SOCl_2_, DCM/DMF, 0 °C–rt, o.n., 2. DIPEA, DCM, r.t., 18.5 h; (e) LiOH, H_2_O/THF, r.t., 4 h; and (f) BBr_3_, DCM, −78 °C–r.t., o.n.

The second designed siderophore-containing MBL inhibitor **3** was obtained starting from 3,4-Dimethoxyphenylpropanoic acid **13**, which was coupled with 1-Boc-protected Piperazine-2-carboxylic acid in a Schotten–Baumann reaction (Scheme 2). The free carboxylate **14** was protected by benzylation to obtain compound **15**, which was subsequently deprotected at N1 by the cleavage of the Boc group under acidic conditions. Mercaptomethylpropionic acid was coupled to the hydrochloride **16**, again under Schotten–Baumann conditions. Deprotection steps yielded the desired compound **3**, while the dimethoxy precursor **18** was also characterised (vide supra).

### 2.3. Experimental Evaluation

The potential siderophore-containing MBL inhibitors **2** and **3,** as well as their dimethoxy counterparts **12** and **18,** were tested in a fluorescence-based enzyme activity assay. The MBLs VIM-1, IMP-7, and NDM-1 were recombinantly expressed in *E. coli* and the enzymatic activity was monitored by the conversion of the fluorogenic substrate fluorocillin [17], as described before [18,19]. All compounds were able to inhibit the selected MBLs in a low micromolar to submicromolar concentration range (Table 1). Interesting trends could be observed regarding compounds **2** and **3** and their dimethoxy counterparts **12** and **18**. The free catechol moiety of compound **2** led to an improvement of inhibitory potency against VIM-1, while NDM-1 and IMP-7 tolerated both compounds, **2** and **12**, equally well. In contrast, the deprotected catechol **3** outperformed the dimethoxy counterpart **18** in the inhibition of all three tested MBLs. Furthermore, in contrast to compound **2**, the catechol inhibitor **3** exhibited submicromolar activity towards all three MBLs, which qualifies it for further evaluation.

**Figure 3:**
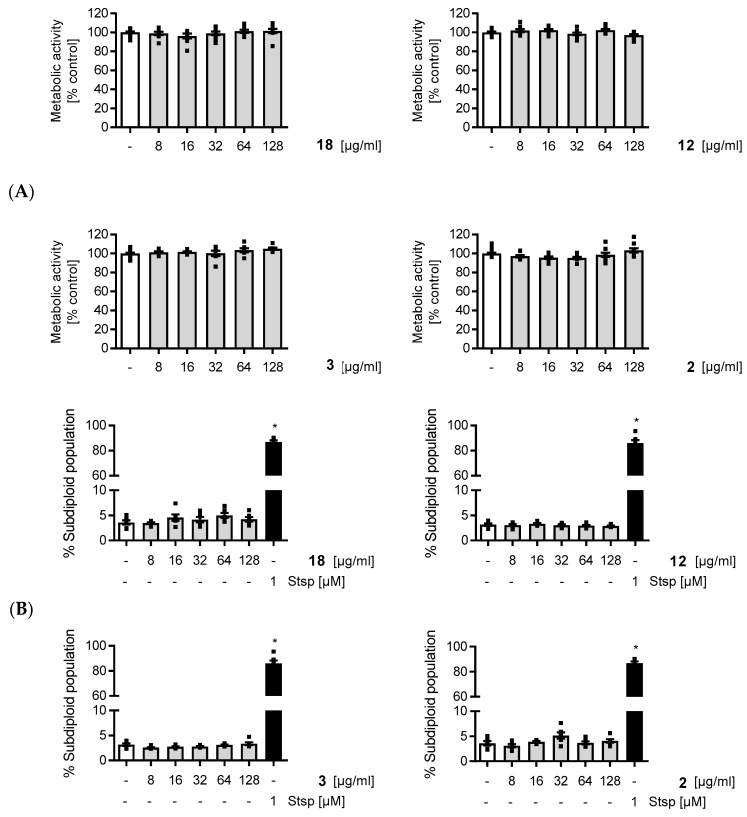
Evaluation of compounds **2**, **3**, **12**, and **18** in cytotoxicity assays. * Staurosporine (Stsp) was used as cytotoxic control.

Complicated infections are often treated by the intravenous application of anti-infectives. Hence, the inner lining of the blood vessels, called endothelium, is highly prone to damages caused by the cytotoxic actions of these drugs. Therefore, we evaluated the potential cytotoxic effects of compounds **2**, **3**, **12,** and **18** in vitro, using primary human umbilical vein endothelial cells (HUVECs). We found that concentrations of up to 128 µg/mL of compounds **2**, **3**, **12,** and **18** neither influenced the cell viability (Figure 3A) nor the apoptosis rate (Figure 3B) of HUVECs in a negative manner.

The considerable inhibition of purified MBLs in vitro, especially of NDM-1, with compound **3** suggested that these MBL inhibitors can potentially restore the activity of imipenem against MBL-expressing bacteria. To prove this, imipenem combined with different concentrations of mjr derivatives ranging from 2 to 128 mg/L was tested against a clinical *K. pneumoniae* isolate expressing NDM-1 (Table 2). The most potent inhibitor, i.e., compound **3**, significantly reduced the MIC of imipenem by 16-fold.

**Figure 4:**
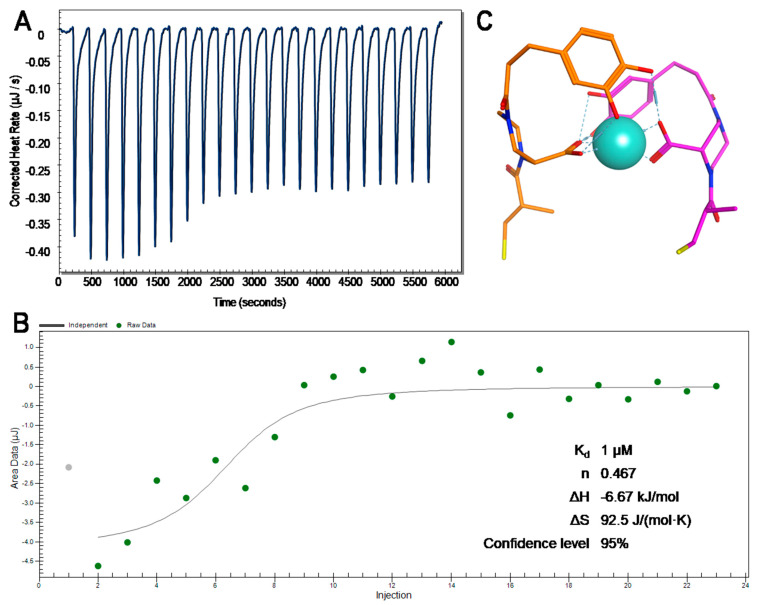
Binding of compound **3** to Fe^3+^. (**A**). ITC measurement of the binding event. FeCl_3_ solution was titrated to the solution of compound **3**. (**B**). Model fit of the **3**:Fe^3+^ binding. (**C**). Model structure of two molecules of 3 (orange and purple sticks) binding to Fe^3+^ ion (cyan sphere).

We investigated the potential of compound **3** to bind Fe^3+^ ions via isothermal titration calorimetry (Figure 4). In this context, we titrated 250 µM of FeCl_3_ to 50 µM of compound **3** and observed a binding event. When fitting the data with a model of independent binding, we observed that n = 0.467 ± 0.112, which approximately corresponds to the stoichiometry of 2:1 for the **3**:Fe^3+^ complex. These findings are consistent with the published data, e.g., 2:1 complexes of siderophores with iron are reported for the *A. baumanii* siderophore acinetobactin [20].

**Figure 5:**
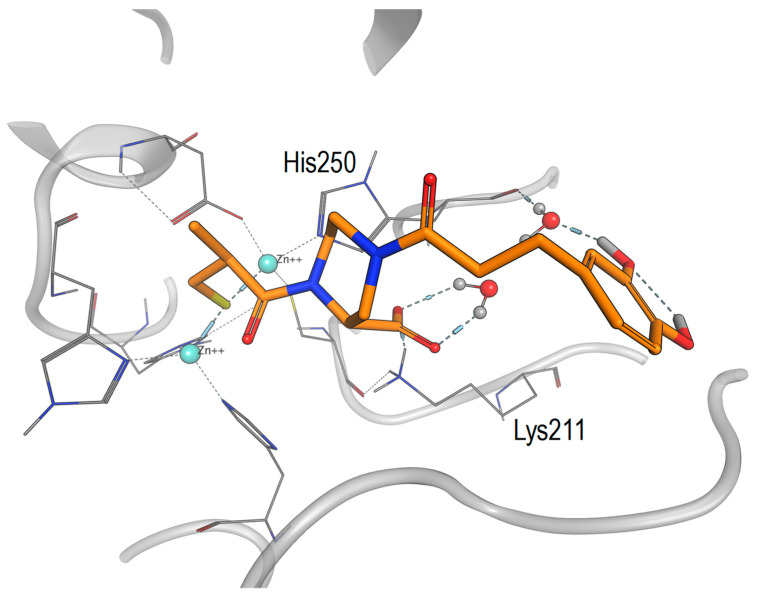
Proposed binding mode of compound **3** to NDM-1. Starting from the X-ray structure of compound **1** in complex with NDM-1 (PDB code 6LJ0), the catechol moiety was introduced manually and the complex was subsequently energy minimised.

We examined the potential binding mode of compound **3** in complex with NDM-1. Therefore, we manually extended the C4-position of the D-pipecolic acid of compound **3** in complex with NDM-1 (PDB code 6LJ0) using the 3,4-dihydroxyphenyl propanoic acid residue, and subsequently performed an energy minimization of the complex (Figure 5). We observed the ionic interactions of the carboxylate moiety bindings towards Lys211, while the catechol moiety extended into a more solvent-accessible binding site, where it formed water-mediated H bonds towards the backbone NH of His250.

## 3. Discussion

In this study we aimed for the design and synthesis of siderophore-containing MBL inhibitors. Compound **2** potently inhibited VIM-1 and IMP-7, however, it failed to inhibit NDM-1 in submicromolar range. Compound **3**, which extends the D-pipecolic acid-based MBL inhibitors, inhibited all tested MBLs in submicromolar range. Thereby, it is more potent than captopril or tiopronin, which exhibited inhibitory values in a low micromolar range in the same activity assay performed in our group [8]. These findings suggest that future studies to design siderophore-containing MBL inhibitors should concentrate on the extension of the D-pipecolic acid in order to elaborate on the structure–activity relationships of this compound class. Furthermore, the efficacy of the siderophore should be improved. Different iron-chelating moieties, which had already served as useful siderophores in the past [21], could be coupled with D-pipecolic acid. Although the iron-chelating affinity of compound **3** can be considered as mediocre, compound **3** exhibited a significant improvement in the MIC of imipenem in a clinical *K. pneumoniae* isolate, expressing NDM-1 at concentration of 16 µg/mL. Compared to compound 58 [5], the adjuvant activity of compound **3** can be further improved. Extensive further characterization of a range of different isolates should be performed in order to evaluate the potential of compound **3**. Taken together, this study is the first report of an MBL inhibitor that contains a siderophore moiety which displays potent inhibitory activity against recombinant MBLs in vitro, and restores the antimicrobial activity of imipenem in a clinical *K. pneumoniae* isolate without affecting primary human cells.

## 4. Materials and Methods

### 4.1. General Information

Reagents and solvents were purchased from the suppliers BLD Pharmtech GmbH (Kaiserslautern, Germany), Sigma Aldrich (Darmstadt, Germany), TCI Europe N.V. (Zwijndrecht, Belgium), or Fluorochem Ltd. (Derbyshire, United Kingdom), and used without further purification. Flash chromatography was performed on packed silica columns (particle size 50 µM) from Interchim (Montlucon Cedex, France) and with solvents at technical grade (for mixtures, see the corresponding experiments). Analytical tin layer chromatography (TLC) was performed with F254 TLC plates from Merck KGaA (Darmstadt, Germany); aromatic systems were visualised with ultraviolet light (240 nm). NMR spectra were measured on an AV 400, AV 500, or DRX 600 nuclear magnetic resonance spectrometer from Bruker (Karlsruhe, Germany). Chemical shifts were reported in parts per million (ppm), using TMS as an external standard, and the residual proton signal of the deuterated solvents (DMSO-d6) were used as an internal standard. The following abbreviations are used for the multiplicity of the signals: singlet (s), doublet (d), triplet (t), quartet (q), multiplet (m), and doublet of doublets (dd). The coupling constant (J) was determined by a machine and expressed in Hertz (Hz). HPLC and mass analysis were performed on a LCMS 2020 from Shimadzu (Duisburg, Germany). For analytical determination, a Luna 10u C18(2) (250 × 4.6 nm) was used, and for semi-preparative purification, a Luna 10μ C18(2) (250 × 21.20 nm) column from Phenomenex LTD Deutschland (Aschaffenburg, Germany) was used. The system is equipped with a SPD 20A UV/VIS detector (λ = 240/280 nm) and an ESI-TOF (measuring in the positive- and/or negative-ion mode). Eluent mixtures of acetonitrile/0.1% aqueous formic acid were used, with a flow rate of 0.1 mL/min (Scout Column) or 21 mL/min (semi-preparative) at room temperature. The following methods were used: method 1 was 0 min 95% ACN, 2 min 95% ACN, 14 min 10% ACN, and 20 min 10% ACN; and method 2 was 0 min 50% ACN, 10 min 10% ACN, and 15 min 10% ACN. The purity of the compounds was determined by integrating the peaks of the UV chromatogram. All compounds were found to be >95% pure through HPLC analysis. Further MS spectra were obtained on a Thermo Fisher Surveyor MSQ coupled with a Camag TLC-MS interface 2. High-resolution mass spectra were recorded on an MALDI LTQ ORBITRAP XL instrument (Thermo Fisher Scientific Inc., Waltham, MA, USA). The matrix used was α-cyano-4-hydroxycinnamic acid (HCCA). The structure of all compounds presented were verified by a least two of the described methods, and the final compounds were verified by NMR, Mass, HRMS and HPLC to determine a purity of >95%.

### 4.2. Synthesis

*5-(2,3-Dimethoxybenzylidene)-2,2-dimethyl-1,3-dioxane-4,6-dione* (**5**). 2,3-Dimethoxybenzaldehyde (**4**, 5.1 g, 29.9 mmol, 0.9 eq) and AcOH (0.2 mL, 3.3 mmol, 0.1 eq) were added to 2-propanol (210 mL). Meldrum acid (4.9 g, 33.2 mmol, 1.0 eq) was added portion-wise. The reaction was stirred at 75 °C for 3 h. After cooling to room temperature, the solvent was removed in vacuo. Purification was carried out using flash chromatography, with silica gel as stationary phase and Hexane/EtOAc (gradient 100/0 to 0/100) as mobile phase. Product **5** was obtained as yellow oil (7.3 g, 66%). ^1^H NMR (400 MHz, DMSO-*d*_6_): δ = 7.32 (d, *J* = 8.1 Hz, 1H), 7.25 (d, *J* = 8.1 Hz, 1H), 7.13 (t, *J* = 8.0 Hz, 1H), 3.85 (s, 3H), 3.79 (s, 3H), 3.31 (s, 1H), 1.77 (s, 6H) ppm. MS (ESI, 70 eV) *m/z* (%): 293.85 (100) (M + H). ^1^H NMR spectra of synthetized compounds can be found in the Supplementary Materials.

*5-(2,3-Dimethoxybenzyl)-2,2-dimethyl-1,3-dioxane-4,6-dione* (**6**). Dione (**5**, 7.2 g, 17.2 mmol, 1.0 eq) was solved into DCM (200 mL) at 0 °C. AcOH (31 mL, 533 mmol, 31 eq) was added and the mixture was stirred at 0 °C for 15 min. NaBH_4_ (5.3 g, 0.1 mol, 8.0 eq) was added in portions at 0 °C and the reaction mixture was stirred at room temperature for one hour. After the decolourisation of the reaction mixture, H_2_O (200 mL) was added and extracted with DCM (3 × 200 mL). The combined organic phases were washed with H_2_O (200 mL), saturated with NaCl solution (200 mL), and dried over MgSO_4_. The solvent was removed to obtain the crude product, which was purified by flash chromatography (silica gel as stationary phase, gradient of DCM/MeOH 100/0 to 90/10 as mobile phase) to obtain the product **6** as a colourless solid (3.0 g, 53%). ^1^H NMR (400 MHz, DMSO-*d*_6_): δ = 6.96 (t, *J* = 7.9 Hz, 1H), 6.89 (d, *J* = 6.7 Hz, 1H), 6.75 (d, *J* = 7.0 Hz, 1H), 4.64 (t, *J* = 5.4 Hz, 1H), 3.78 (s, 3H), 3.72 (s, 3H), 3.29 (d, *J* = 5.4 Hz, 2H), 1.82 (s, 3H), 1.68 (s, 3H) ppm. MS (ESI, 70 eV) *m/z* (%): 294.90 (100) (M + H).

*Methyl 2-(2,3-dimethoxybenzyl)acrylate* (**7**). Dione (**6**, 3.0 g, 10.1 mmol, 1.0 eq) and Eschenmoser’s salt (6.7 mg, 35.2 mmol, 3.5 eq) were dissolved in MeOH (160 mL). The reaction mixture was stirred at 70 °C for 18 h. After cooling to room temperature, the solvent was removed in vacuo and the residue was taken up in DCM. The organic phase was washed with saturated NaHCO_3_ solution (2 × 80 mL) and 10% NaHSO_4_ solution (2 × 80 mL), and the aqueous phase was extracted with DCM (2 × 80 mL). The combined organic phases were washed with saturated NaCl solution (80 mL) and dried over MgSO_4_. After removal of the solvent in vacuo, the crude product was purified by flash chromatography (gradient hexane/EtOAc 96/4 to 0/100) to isolate the product **7** as a colourless oil (1.7 g, 67%). ^1^H NMR (400 MHz, DMSO-*d*_6_) δ = 7.01–6.96 (m, 1H), 6.92 (dd, *J* = 8.2, 1.7 Hz, 1H), 6.69 (dd, *J* = 7.6, 1.6 Hz, 1H), 6.14 (q, *J* = 1.1 Hz, 1H), 5.41 (q, *J* = 1.5 Hz, 1H), 3.79 (s, 3H), 3.68 (s, 3H), 3.67 (s, 3H), 3.56 (t, *J* = 1.2 Hz, 2H) ppm. MS (ESI, 70 eV) *m/z* (%): 237.15 (100) (M + H).

*2-(2,3-Dimethoxybenzyl)acrylic acid* (**8**). Acrylate (**7**, 1.7 g, 6.4 mmol, 1.0 eq) was dissolved in NaOH (1 M, 20 mL) and stirred under microwave irradiation at 100 °C for 1 h. The reaction mixture was washed with diethyl ether (2 × 20 mL) and the aqueous phase was adjusted to pH 1 with HCl (2 M, 13 mL). Subsequently, the reaction mixture was extracted with diethyl ether (3 × 20 mL) and the combined organic phases were dried over MgSO_4_ and in vacuo. The resulting crude product of **8** was obtained as a colourless oil (0.8 mg, 54%) and used without further purification. ^1^H NMR (400 MHz, DMSO-*d*_6_): δ = 12.49 (s, 1H), 7.01–6.96 (m, 1H), 6.91 (dd, *J* = 8.2, 1.6 Hz, 1H), 6.70 (dd, *J* = 7.6, 1.6 Hz, 1H), 6.13–6.07 (m, 1H), 5.34 (q, *J* = 1.6 Hz, 1H), 3.79 (s, 3H), 3.68 (s, 3H), 3.53 (s, 2H) ppm. MS (ESI, 70 eV) *m/z* (%): 220.75 (100) (M–H).

*tert-Butyl (R)-piperidine-2-carboxylate* (**19**). D-Pipecolic acid (2.0 g, 15.0 mmol, 1.0 eq) was suspended in *tert*-butyl acetate (0.1 L, 0.7 mol, 47.0 eq). Under ice bath cooling, 60% perchloric acid (2.3 mL, 24.0 mmol, 1.6 eq) was added dropwise and the reaction mixture was stirred overnight at room temperature. The reaction mixture was extracted with water (3 × 90 mL) and saturated NaHCO_3_ solution was added to the combined aqueous phases until gas development stopped. The aqueous phase was extracted with EtOAc (5 × 45 mL), the combined organic phases were washed with NaCl solution, dried over Na_2_SO_4_, and the solvent was removed in vacuo. Product **19** was obtained as a yellowish solid (1.0 g, 48%) and used without further purification. ^1^H NMR (400 MHz, DMSO-*d*_6_): δ = 3.24 (dd, *J* = 9.7, 3.4 Hz, 1H), 2.95 (dtd, *J* = 12.0, 3.8, 1.3 Hz, 1H), 2.58–2.53 (m, 1H), 2.53–2.51 (m, 1H), 1.82–1.75 (m, 1H), 1.70–1.64 (m, 1H), 1.50–1.43 (m, 1H), 1.41 (s, 9H), 1.40–1.28 (m, 3H) ppm. MS (ESI, 70 eV) *m/z* (%): 185.90 (100) (M + H).

*tert-Butyl (R)-1-(2-(2,3-dimethoxybenzyl)acryloyl)piperidine-2-carboxylate* (**9**). SOCl_2_ (0.6 mL, 8.3 mmol, 2.5 eq) was solved in DCM (30 mL), together with a drop of DMF. Acrylic acid (**8**, 0.8 g, 3.3 mmol, 1.0 eq) was added and the resulting mixture was stirred for 3 h at 50 °C. After the removal of the excess SOCl_2_, the resulting acid chloride was suspended in 1,2-dichloroethane (19 mL) and protected *D*-pipecolic acid (**19**, 0.8 mg, 3.6 mmol, 1.1 eq). DIPEA (1.7 mL, 9.9 mmol, 3.0 eq) was added slowly to the suspension. The reaction was stirred under microwave irradiation for 1 h at 90 °C. The mixture was diluted with water (40 mL), the aqueous phase was acidified with HCl (2 M), and extracted with EtOAc (3 × 20 mL). The combined organic phases were washed with HCl (2 M, 3 × 20 mL) and saturated NaCl solution (17 mL), and dried over MgSO_4_. After the removal of all solvents in vacuo, the crude product was obtained. Purification was performed using flash chromatography with silica gel as stationary phase and a mixture of hexane/EtOAc (gradient 100/0 to 0/100) as mobile phase, to isolate product **9** as a yellow oil (1.1 g, 63%). ^1^H NMR (400 MHz, DMSO-*d*_6_) δ = 7.01 (t, *J* = 7.8 Hz, 1H), 6.94 (d, *J* = 8.4 Hz, 1H), 6.74 (t, *J* = 7.8 Hz, 1H), 5.05–4.80 (m, 3H), 3.79 (s, 3H), 3.70 (s, 3H), 3.51–3.43 (m, 2H), 3.03 (t, *J* = 13.0 Hz, 1H), 2.61 (t, *J* = 12.5 Hz, 1H), 2.02 (dd, *J* = 54.8, 13.2 Hz, 2H), 1.64–1.56 (m, 1H), 1.41 (s, 9H), 1.27–0.95 (m, 3H) ppm. MS (ESI, 70 eV) *m z* (%): 390.05 (100) (M + H).

*tert-Butyl (2R)-1-(3-(acetylthio)-2-(2,3-dimethoxybenzyl)propanoyl)piperidine-2-carboxylate* (**10**). Carboxylate (**9**, 1.0 g, 2.7 mmol, 1.0 eq) was dissolved in thioacetic acid (40 mL, 535 mmol, 200 eq) and the mixture was stirred at room temperature for 70 h. The mixture was then diluted with water (70 mL) and extracted with EtOAc (3 × 50 mL). The combined organic phases were washed with saturated NaCl solution (60 mL) and dried over MgSO_4_. The solvents were removed in vacuo and the crude product was purified using flash chromatography with silica gel as stationary phase and a mixture of hexane/EtOAC (gradient) as mobile phase, to isolate product **10** as a dark red oil (1.0 g, 72%). ^1^H NMR (400 MHz, DMSO-*d*_6_): δ = 6.98–6.91 (m, 2H), 6.69 (td, *J* = 6.7, 2.4 Hz, 1H), 5.10–4.98 (m, 1H), 3.79 (s, 3H), 3.74 (s, 3H), 3.27–3.08 (m, 2H), 3.02–2.87 (m, 2H), 2.74 (d, *J* = 7.4 Hz, 1H), 2.27 (s, 3H), 2.01 (s, 2H), 1.72–1.62 (m, 2H), 1.55–1.45 (m, 2H), 1.41 (s, 9H), 1.32–1.22 (m, 2H) ppm. MS (ESI, 70 eV) *m/z* (%): 466.40 (100) (M + H).

*(2R)-1-(3-(acetylthio)-2-(2,3-dimethoxybenzyl)propanoyl)piperidine-2-carboxylic acid* (**11**). Carboxylate (**10**, 1.0 g, 2.1 mmol, 1.0 eq) was dissolved in DCM (35 mL), and TFA (8.8 mL, 0.1 mol, 57.0 eq) was slowly added by stirring. The reaction mixture was stirred overnight at 50 °C and the solvent was removed under reduced pressure afterwards. After co-evaporation with toluene, **11** was obtained as a crude product. After washing the crude product with hexane (8 × 15 mL), **11** was obtained as a red, highly viscous oil (0.9 g, 83%). ^1^H NMR (400 MHz, DMSO-d6): δ = 12.73 (s, 1H), 7.00–6.90 (m, 2H), 6.81–6.65 (m, 1H), 5.10 (dd, *J* = 25.7, 5.6 Hz, 1H), 3.79 (d, *J* = 4.4 Hz, 3H), 3.76–3.72 (m, 3H), 3.31–3.05 (m, 2H), 2.97–2.83 (m, 2H), 2.74 (d, *J* = 7.0 Hz, 1H), 2.31–2.21 (m, 3H), 2.07 (dd, *J* = 42.8, 13.9 Hz, 2H), 1.76–1.61 (m, 2H), 1.57–1.43 (m, 2H), 1.39–1.13 (m, 2H) ppm. MS (ESI, 70 eV) *m/z* (%): 409.90 (100) (M + H).

*(2R)-1-(2-(2,3-dimethoxybenzyl)-3-mercaptopropanoyl)piperidine-2-carboxylic acid* (**12**, **mjr344**). Acid (**11**, 0.9 mg, 1.7 mmol, 1.0 eq) was added to water (40 mL) and degassed under argon in an ultrasonic bath for 30 min. The solution was cooled to 0 °C and NH_3_ solution (25% in water, 6.5 mL, 45.0 eq) was added. The mixture was stirred for 2 h at room temperature. The reaction was terminated by adding HCl (2 M) and adjusted to pH < 3, extracted with EtOAc (3 × 20 mL), and the combined organic phases were washed with saturated NaCl solution (20 mL) and dried over MgSO_4_. All volatile components were removed in vacuo to obtain **15** as crude product. Purification was carried out using preparative HPLC (C18 column, mobile phase: Iso 42% ACN) to isolate product **12** as a colourless solid (0.2 g, 34%). ^1^H NMR (500 MHz, DMSO-*d*_6_): δ = 12.80 (s, 1H), 7.00–6.88 (m, 2H), 6.79–6.68 (m, 1H), 5.14 (ddd, *J* = 35.7, 6.1, 2.2 Hz, 1H), 4.04 (d, *J* = 13.5 Hz, 1H), 3.79 (d, *J* = 3.2 Hz, 3H), 3.76–3.73 (m, 3H), 3.30–3.15 (m, 2H), 3.14–3.02 (m, 2H), 2.85 (dd, *J* = 13.6, 4.9 Hz, 1H), 2.12 (dd, *J* = 10.1, 6.9 Hz, 2H), 1.71–1.61 (m, 2H), 1.53–1.45 (m, 2H), 1.32–1.23 (m, 2H) ppm. ^13^C NMR (126 MHz, DMSO): δ = 172.66, 172.45, 146.83, 146.71, 132.01, 123.69, 122.54, 111.55, 60.07, 55.67, 51.38, 44.93, 42.90, 32.45, 26.54, 25.18, 20.78, 20.57 ppm. MS (ESI, 70 eV) *m/z* (%): 367.95 (100) (M + H). HRMS calculated *m/z* 368.15262 andfound *m/z* 368.15317.

*(2R)-1-(2-(2,3-dihydroxybenzyl)-3-mercaptopropanoyl)piperidine-2-carboxylic acid* (**2**, **mjr347**). Acid (**12**, 0.16 mg, 0.42 mmol, 1.00 eq) was dissolved in DCM (3 mL) at −78 °C. BBr_3_ (1 M in DCM, 2.09 mL, 2.09 mmol, 5.00 eq) was added, stirred for 2 h at −78 °C and then overnight at room temperature. The resulting mixture was cooled to 0 °C and water was added dropwise until the remaining BBr_3_ was consumed (no more smoke was produced). The aqueous phase was extracted with DCM (3 × 6 mL), the organic phases were combined and all volatile components were removed under reduced pressure to obtain the crude product. The purification was done using preparative HPLC (C18 column, mobile phase: Iso 34% ACN) to isolate product **2/mjr347** as a colourless glass (0.04 g, 28%). ^1^H NMR (500 MHz, DMSO-*d*_6_): δ = 12.72 (s, 1H), 9.22 (d, *J* = 11.8 Hz, 1H), 8.43–8.22 (m, 1H), 6.68–6.62 (m, 1H), 6.55–6.51 (m, 1H), 6.50 (d, *J* = 4.4 Hz, 1H), 5.14 (ddd, *J* = 37.3, 6.0, 2.3 Hz, 1H), 4.11 (d, *J* = 13.7 Hz, 1H), 3.29–3.11 (m, 2H), 3.03 (td, *J* = 13.1, 3.0 Hz, 2H), 2.87–2.71 (m, 1H), 2.34–2.10 (m,2H), 1.72–1.59 (m, 2H), 1.58–1.38 (m, 2H), 1.35–1.07 (m, 2H) ppm. ^13^C NMR (126 MHz, DMSO): δ = 172.97, 172.42, 145.03, 143.33, 125.56, 121.43, 118.56, 113.75, 51.33, 44.56, 42.82, 34.29, 26.57, 25.21, 20.86, 20.54 ppm. MS (ESI, 70 eV) *m/z* (%): 339.95 (100) (M + H).HRMS calculated *m/z* 362.10326 and found *m/z* 362.10352. HPLC: t_R_: 11.56 min (method 1), purity >95%.

*(R)-1-(tert-butoxycarbonyl)-4-(3-(3,4-dimethoxyphenyl)propanoyl)piperazine-2-carboxylic acid* (**14**). 3-(3,4-dimethoxyphenyl)-propionic acid (**13**, 3.4 g, 16.2 mmol, 1.0 eq) was dissolved in SOCl_2_ (50 mL) and stirred for 3 h at 90 °C. After removing the excess SOCl_2_ in vacuo, the obtained acid chloride was mixed with (R)-1-boc-piperazine-2-carboxylic acid (5.0 g, 21.0 mmol, 1.3 eq) in DCM (160 mL), pyridine (20 mL), and DMF (2 mL), and the suspension was stirred for 19 h at room temperature. HCl (10%, 200 mL) was added and the aqueous phase was extracted with EtOAc (3 × 80 mL). The purified organic phases were dried over MgSO_4_ and in vacuo to obtain the crude product. The crude product was purified using flash column chromatography with silica gel as stationary phase and DCM/MeOH +1%AcOH (gradient DCM/MeOH 100/0 to 90/10) as mobile phase, to obtain product **14** as a beige foamy solid (2.5 g, 29%) after drying. ^1^H NMR (400 MHz, DMSO-*d*_6_): δ = 12.94 (s, 1H), 6.85–6.80 (m, 2H), 6.71 (dd, *J* = 8.2, 1.9 Hz, 1H), 4.63–4.40 (m, 1H), 3.91–3.78 (m, 2H), 3.73 (s, 3H), 3.70 (s, 3H), 3.19–2.79 (m, 4H), 2.75–2.67 (m, 2H), 2.66–2.54 (m, 2H), 1.39 (d, *J* = 15.8 Hz, 9H) ppm. MS (ESI, 70 eV) *m/z* (%): 422.95 (100) (M + H).

*2-Benzyl 1-(tert-butyl) (R)-4-(3-(3,4-dimethoxyphenyl)propanoyl)piperazine-1,2-dicarboxylate* (**15**). Acid (**14**, 2.4 g, 4.9 mmol, 1.0 eq), benzyl bromide (0.7 mL, 5.9 mmol, 1.2 eq), and KHCO_3_ (0.7 g, 7.3 mmol,1.5 eq) were suspended in acetone (40 mL) and refluxed for 18 h at 65 °C. Acetone was removed in vacuo and the reaction was diluted with DCM (60 mL) and water (60 mL). The phases were separated and the aqueous phase was extracted with DCM (2 × 60 mL), acidified, and extracted again with EtOAc (2 × 60 mL). The combined organic phases were dried over MgSO_4_ and in vacuo to obtain the crude product. Purification was performed using flash chromatography with silica gel as stationary phase and a mixture of hexane/EtOAc (gradient 87/13 to 0/100) as mobile phase. After drying, product **15** was isolated as an orange oil (2.2 g, 67%). ^1^H NMR (600 MHz, DMSO-d6): δ = 7.37–7.27 (m, 5H), 6.84–6.78 (m, 2H), 6.73–6.63 (m, 1H), 5.17–5.03 (m, 2H), 4.82–4.58 (m, 1H), 3.86–3.75 (m, 2H), 3.71 (dd, *J* = 13.6, 10.6 Hz, 6H), 3.19–2.83 (m, 4H), 2.74–2.64 (m, 2H), 2.63–2.54 (m, 2H), 1.41–1.28 (m, 9H) ppm. MS (ESI, 70 eV) *m/z* (%): 512.80 (100) (M + H).

*Benzyl (R)-4-(3-(3,4-dimethoxyphenyl)propanoyl)piperazine-2-carboxylate* (**16**). Carboxylate (**15**, 2.1 g, 3.3 mmol, 1.0 eq) was dissolved in DCM (50 mL). HCl in dioxane (4 M, 3.3 mL, 13.3 mmol, 4.0 eq) was added, and the mixture was stirred at room temperature overnight. All volatiles were removed in vacuo to obtain product **16** as a colourless foamy solid (1.8 g., quant.), which was used without further purification. ^1^H NMR (600 MHz, DMSO-*d*_6_): δ = 7.46–7.35 (m, 5H), 6.85–6.80 (m, 2H), 6.71 (dd, *J* = 24.6, 8.2 Hz, 1H), 5.25 (s, 2H), 4.50–4.23 (m, 1H), 3.74–3.70 (m, 6H), 3.56 (s, 1H), 3.25 (td, *J* = 10.5, 9.7, 5.1 Hz, 2H), 3.05–2.92 (m, 4H), 2.72 (q, *J* = 7.6 Hz, 2H), 2.65 (dd, *J* = 9.8, 6.8 Hz, 2H) ppm. MS (ESI, 70 eV) *m/z* (%): 412.85 (100) (M + H).

*Benzyl (R)-1-((S)-3-(acetylthio)-2-methylpropanoyl)-4-(3-(3,4-dimethoxyphenyl)propanoyl)piperazine-2-carboxylate* (**17**). (S)-(-)-3-acethylthio)-2-methylpropionic acid (4.1 g, 25.1 mmol, 1.0 eq) was dissolved in DCM (100 mL + 0.1 mL DMF), and SOCl_2_ (2.4 mL, 32.6 mmol, 1.3 eq) was added at 0 °C and the mixture was stirred at room temperature overnight. The solvent was removed in vacuo to obtain the acid chloride. Amine (**16**, 1.2 g, 2.7 mmol, 1.0 eq) was dissolved in DCM (40 mL), and DIPEA (0.7 mL, 4.0 mmol, 1.5 eq) was added and the mixture was stirred for 30 min at room temperature. Acid chloride (0.5 g, 2.7 mmol, 1.0 eq) was added and the mixture stirred overnight at room temperature. The reaction mixture was washed with HCl (2 M, 40 mL), as well as NaOH (1 M, 40 mL) and saturated sodium chloride solution (40 mL). The organic phase was dried in vacuo to obtain **17** as a crude product. The purification was performed using flash chromatography with silica gel as stationary phase and a mixture of DCM/EtOAc (gradient 100/0 to 0/100) as mobile phase. After drying, product **17** was isolated as a yellow oil (1.4 g, 75%). ^1^H NMR (600 MHz, DMSO-*d*_6_): δ = 7.38–7.27 (m, 5H), 6.85–6.78 (m, 2H), 6.68 (dd, *J* = 34.6, 8.2 Hz, 1H), 5.18–4.99 (m, 2H), 4.38–4.21 (m, 1H), 3.94–3.78 (m, 2H), 3.75–3.67 (m, 6H), 3.33–3.14 (m, 4H), 3.05–2.93 (m, 3H), 2.85–2.78 (m, 2H), 2.62–2.53 (m, 2H), 2.33–2.29 (m, 3H), 1.13–1.07 (m, 3H) ppm. MS (ESI, 70 eV) *m/z* (%): 557.00 (100) (M + H).

*(R)-4-(3-(3,4-dimethoxyphenyl)propanoyl)-1-((S)-3-mercapto-2-methylpropanoyl)piperazine-2-carboxylic acid* (**18**, **mjr324**). LiOH (0.2 g, 7.9 mmol, 4.5 eq) was dissolved in water (120 mL) and THF (60 mL) and carboxylate (**17**, 1.0 g, 1.8 mmol, 1 eq) were added. The suspension was stirred for 4.5 h at room temperature. HCl (2 M, 200 mL) was added and the mixture was extracted with DCM (3 × 60 mL). Purification was performed using preparative HPLC (C18 column, mobile phase: Iso 42% ACN) to obtain the product (**18**, **mjr324**) as a colourless foam (0.4 g, 47%). ^1^H NMR (500 MHz, DMSO-*d*_6_): δ = 12.96 (s, 1H), 6.86–6.81 (m, 2H), 6.72 (dd, *J* = 8.1, 2.3 Hz, 1H), 4.87 (dd, *J* = 5.0, 2.3 Hz, 1H), 4.37–4.24 (m, 1H), 3.97–3.79 (m, 2H), 3.73 (s, 3H), 3.70 (s, 3H), 3.32–3.10 (m, 4H), 3.05–2.87 (m, 2H), 2.79–2.68 (m, 3H), 2.66–2.56 (m, 2H), 1.13–0.97 (m, 3H) ppm. ^13^C NMR (126 MHz, DMSO): δ = 174.14, 171.08, 170.13, 148.63, 147.09, 133.74, 120.00, 112.34, 111.94, 55.57, 45.98, 45.57, 45.40, 42.24, 41.22, 38.75, 33.94, 30.18, 16.96 ppm. MS (ESI, 70 eV) *m/z* (%): 425.30 (100) (M + H). HRMS calculated *m/z* 447.15603 and found *m/z* 447.15561.

*(R)-4-(3-(3,4-dihydroxyphenyl)propanoyl)-1-((S)-3-mercapto-2-methylpropanoyl)piperazine-2-carboxylic acid* (**3**, **mjr345**). Acid (**3**, 0.3 g, 0.7 mmol, 1.0 eq) was dissolved in DCM (3 mL) at −78 °C. BBr_3_ (1 M in DCM, 3.5 mL, 3.5 mmol, 5.0 eq) was added. The resulting mixture was stirred for 2 h at −78 °C and then overnight at room temperature. The reaction mixture was cooled to 0 °C and water was added dropwise until the remaining BBr_3_ was consumed (no more smoke was produced). The aqueous phase was extracted with DCM (3 × 6 mL), the organic phases were combined, and all volatile components were removed under reduced pressure to obtain the crude product. Purification was performed using preparative HPLC (C18 column, mobile phase: Iso 26% ACN) to obtain the product **3**, **mjr345** as a colourless solid (20.0 mg, 7%). ^1^H NMR (500 MHz, DMSO-*d*_6_): δ = 13.18 (s, 1H), 8.63 (s, 2H), 6.61 (dd, *J* = 6.6, 3.9 Hz, 1H), 6.59 (d, *J* = 2.0 Hz, 1H), 6.44 (dd, *J* = 8.0, 2.1 Hz, 1H), 5.07–4.66 (m, 1H), 4.28 (q, *J* = 12.0, 11.2 Hz, 1H), 3.98–3.76 (m, 2H), 3.34–3.13 (m, 4H), 3.04–2.88 (m, 2H), 2.77–2.68 (m, 1H), 2.65–2.56 (m, 4H), 1.13–0.96 (m, 3H) ppm. ^12^C NMR (126 MHz, DMSO): δ = 174.09, 171.27, 171.04, 144.96, 143.32, 131.99, 118.75, 115.74, 115.42, 45.94, 45.37, 42.72, 42.21, 41.92, 38.79, 34.11, 29.97, 16.90 ppm. MS (ESI, 70 eV) *m/z* (%): 396.95 (100) (M + H). HRMS calculated *m/z* 419.12473 and found *m/z* 419.12439.

### 4.3. Enzymatic Activity Assays

The enzymatic activity assays relied on the cleavage of the fluorogenic substrate fluorocillin with the recombinant MBLs [8]. Fluorocillin was synthetised, as described by Rukavishnikov et al. [17]. The MBLs VIM-1, IMP-7, and NDM-1 were recombinantly expressed in *E. coli* and were diluted in an assay buffer (HEPES 50 mM, pH 7.5; 0.01 %Triton X-100). The final protein concentration of the MBLs and supplemented ZnCl_2_ was: VIM-1–4 nM, IMP-7–0.1 nM, and NDM-1–3 nM. 1 µL of the inhibitors dissolved in DMSO was added to 89 µL of the protein solution. After 30 min of incubation with the inhibitors, 10 µL of fluorocillin solution in an assay buffer (888 nM fluorocillin, HEPES 50 mM, pH 7.5; 0.01 %Triton X-100) was added (100 µL final assay volume; 1% final DMSO concentration) and the fluorescence gain was measured for 30 cycles using a Tecan fluorescent plate reader (Infinite F200; excitation at 495 nm and emission at 525 nm). Negative controls were measured in the absence of enzymes (89 µL of assay buffer), whereas the positive controls were measured in the presence of enzymes and in the absence of inhibitors (1 µL of DMSO). The inhibitory effect of each substance was measured in triplicate in three independent experiments. IC_50_ values were calculated using the data obtained from measurements with at least eight different inhibitor concentrations, applying a sigmoidal dose–response (variable slope with four parameters) equation using GraphPad Prism 5 (GraphPad Software, La Jolla, CA, USA) software.

### 4.4. ITC Measurements

ITC experiments were performed using an Affinity ITC (TA Instruments, Eschborn, Germany), which operated at constant temperature of 25°C. The complexation experiment was carried out with the most potent compound, compound **3** (50 µM in H_2_O_dest._, containing 1% *(v/v*) DMSO), which was placed in the cell., while the syringe contained 250 µM of FeCl_3_ (in H_2_O_dest._) and 1% (*v*/*v*) DMSO). The titration was performed with 23 injections (injection volume of 2.5 µL) and an injection interval of 250 s. Corresponding blank measurements with H_2_O(dest.) and 1% (*v/v*) DMSO in the cell or the syringe were performed. Experimental data were analyzed using software “NanoAnalyze Data Analysis” (Version 3.12.5, TA Instruments) by subtracting a fix heat, determined by the blank experiments and using the “independent” fit option for evaluating the data.

### 4.5. Cytotoxicity Measurements

Compounds **2**, **3**, **12**, and **18** were dissolved in dimethyl sulfoxide (DMSO; Sigma-Aldrich, St. Louis, MO, USA) to a stock solution of 128 mg/mL. Staurosporine (Stsp; Biomol, Hamburg, Germany) was dissolved in DMSO to a stock solution of 1 mM. The stock solutions were stored at −20 °C and were freshly diluted for experimental purposes using cell culture medium, without exceeding a final DMSO concentration of 0.1% (*v/v*).

Human umbilical vein endothelial cells (HUVECs) were isolated from human umbilical veins, as previously described [22,23]. These HUVECs were cultivated to passage 2 and used for experimental purposes in passage 3.

To measure the metabolic activity of the HUVECs treated with compound **2**, **3**, **12**, and **18**, the CellTiter-Blue cell viability assay (Promega, Mannheim, Germany) was used, according to the manufacturer’s instructions. In brief, the HUVECs were treated as indicated for 24 h. Four hours before the end of the incubation period, the CellTiter Blue reagent was added to the cells (1:10 dilution). The fluorescence intensity was measured using a microplate reader (SPECTRAFluor Plus; Tecan, Männedorf, Switzerland) at 535 nm (ex) and 590 nm (em).

To analyse the influence of compounds **2**, **3**, **12**, and **18** on the late apoptosis of HUVECs, propidium iodide (PI) staining, according to Nicoletti et al., was conducted [24]. The HUVECs were treated as indicated for 24 h. Subsequently, cell culture supernatants were collected, and the HUVECs were detached using a trypsin/EDTA solution (Biochrom). The cells were washed with ice-cold PBS and incubated with a solution containing 50 µg/mL PI (Sigma-Aldrich, St. Louis, MO, USA), 0.1% Triton X-100 (Sigma-Aldrich), and 0.1% sodium citrate (Carl Roth, Karlsruhe, Germany) overnight at 4 °C in the dark. For the induction of apoptosis, 1 µM stsp served as a positive control. The percentage of cells with sub-diploid DNA content was analysed by recording the median values for 10,000 events per sample using a FACSVerse flow cytometer (BD Biosciences, San Jose, CA, USA).

Data are expressed as the mean ± standard error of the mean (SEM). The number of independently performed experiments (*n*) indicates different donors for the HUVECs. The actual number of performed experiments is stated in the respective figure legends. Statistical analyses were performed using GraphPad Prism software version 7.0 (San Diego, CA, USA). A one-way ANOVA followed by Tukey’s post hoc test were used for the evaluation of significant differences. *p* ≤ 0.05 was considered as statistically significant.

### 4.6. Isolate Testing

The minimal inhibitory concentrations (MICs) of imipenem monohydrate (Sigma-Aldrich, Steinheim, Germany) ± compounds **2**, **3**, **12**, and **18**, against a clinical *K. pneumoniae* isolate producing NDM-1 carbapenemase, were determined according to the microdilution method established by the Clinical and Laboratory Standards Institute (CLSI).

### 4.7. Molecular Modelling Studies

The X-ray structure of compound 1 in complex with NDM-1 (PDB code 6LJ0; [16]), was prepared for molecular modelling using the QuickPrep routine of the MOE2019.0102 software (Chemical Computing Group; Montreal, QC, Canada), which included the modelling of unresolved loops and the adjustment of the protonation state, as well as energy minimization. The catechol moiety was introduced manually using the builder tool, and the complex was subsequently energy minimised.

## Figures and Tables

**Table 1:** IC_50_ values ± SD [µM] of compounds **2**, **3**, **12**, and **18** obtained in enzymatic fluorescence-based assay with recombinant β-lactamases VIM-1, IMP-7, and NMD-1. All measurements were performed in at least triplicates.

Compound	VIM-1	IMP-7	NDM-1
**12**	1.3 ± 0.02	0.27 ± 0.018	1.9 ± 0.27
**2**	0.17 ± 0.021	0.36 ± 0.052	4.3 ± 0.44
**18**	1.3 ± 0.31	0.11 ± 0.021	0.22 ± 0.016
**3**	0.42 ± 0.024	0.045 ± 0.009	0.13 ± 0.017

**Table 2:** Effects of **2**, **3**, **12**, and **18** at various concentrations on MICs of imipenem for the clinical *K. pneumoniae* isolate expressing NDM-1.

Compound Concentration [µg/mL]	12	2	18	3
0	32	32	32	32
128	8	8	8	2
64	16	16	8	4
32	16	16	16	8
16	32	16	32	8
8	32	32	32	16
4	32	32	32	32
2	32	32	32	32

## Data Availability

Not applicable.

## Supplementary Materials

The following supporting information can be downloaded at: https://www.mdpi.com/article/10.3390/molecules28041984/s1, ^1^H-NMR spectra of synthetized compounds.

## Sample Availability

Samples of the compounds **2**, **3**, **12**, and **18** available from the authors upon request.

## References

[1] Li X., Zhao J., Zhang B., Duan X., Jiao J., Wu W., Zhou Y., Wang H. (2022). Drug Development Concerning Metallo-β-Lactamases in Gram-Negative Bacteria. Front. Microbiol..

[2] Tooke C.L., Hinchliffe P., Bragginton E.C., Colenso C.K., Hirvonen V.H.A., Takebayashi Y., Spencer J. (2019). β-Lactamases and β-Lactamase Inhibitors in the 21st Century. J. Mol. Biol..

[3] Principe L., Vecchio G., Sheehan G., Kavanagh K., Morroni G., Viaggi V., di Masi A., Giacobbe D.R., Luzzaro F., Luzzati R. (2020). Zinc Chelators as Carbapenem Adjuvants for Metallo-β-Lactamase-Producing Bacteria: In Vitro and In Vivo Evaluation. Microb. Drug Resist..

[4] Krajnc A., Lang P.A., Panduwawala T.D., Brem J., Schofield C.J. (2019). Will Morphing Boron-Based Inhibitors Beat the β-Lactamases?. Curr. Opin. Chem. Biol..

[5] Chen C., Oelschlaeger P., Wang D., Xu H., Wang Q., Wang C., Zhao A., Yang K.-W. (2022). Structure and Mechanism-Guided Design of Dual Serine/Metallo-Carbapenemase Inhibitors. J. Med. Chem..

[6] Brem J., Panduwawala T., Hansen J.U., Hewitt J., Liepins E., Donets P., Espina L., Farley A.J.M., Shubin K., Campillos G.G. (2022). Imitation of β-Lactam Binding Enables Broad-Spectrum Metallo-β-Lactamase Inhibitors. Nat. Chem..

[7] Li R., Chen X., Zhou C., Dai Q.-Q., Yang L. (2022). Recent Advances in β-Lactamase Inhibitor Chemotypes and Inhibition Modes. Eur. J. Med. Chem..

[8] Klingler F.-M., Wichelhaus T.A., Frank D., Cuesta-Bernal J., El-Delik J., Müller H.F., Sjuts H., Göttig S., Koenigs A., Pos K.M. (2015). Approved Drugs Containing Thiols as Inhibitors of Metallo-β-Lactamases: Strategy To Combat Multidrug-Resistant Bacteria. J. Med. Chem..

[9] Grigorenko V.G., Khrenova M.G., Andreeva I.P., Rubtsova M.Y., Lev A.I., Novikova T.S., Detusheva E.V., Fursova N.K., Dyatlov I.A., Egorov A.M. (2022). Drug Repurposing of the Unithiol: Inhibition of Metallo-β-Lactamases for the Treatment of Carbapenem-Resistant Gram-Negative Bacterial Infections. Int. J. Mol. Sci..

[10] Büttner D., Kramer J.S., Klingler F.-M., Wittmann S.K., Hartmann M.R., Kurz C.G., Kohnhäuser D., Weizel L., Brüggerhoff A., Frank D. (2018). Challenges in the Development of a Thiol-Based Broad-Spectrum Inhibitor for Metallo-β-Lactamases. ACS Infect. Dis..

[11] Kramer J., Özkaya Ö., Kümmerli R. (2020). Bacterial Siderophores in Community and Host Interactions. Nat. Rev. Microbiol..

[12] Górska A., Sloderbach A., Marszałł M.P. (2014). Siderophore–Drug Complexes: Potential Medicinal Applications of the ‘Trojan Horse’ Strategy. Trends Pharmacol. Sci..

[13] Braun V., Pramanik A., Gwinner T., Köberle M., Bohn E. (2009). Sideromycins: Tools and Antibiotics. Biometals.

[14] McCreary E.K., Heil E.L., Tamma P.D. (2021). New Perspectives on Antimicrobial Agents: Cefiderocol. Antimicrob. Agents Chemother..

[15] El-Lababidi R.M., Rizk J.G. (2020). Cefiderocol: A Siderophore Cephalosporin. Ann. Pharmacother..

[16] Ma G., Wang S., Wu K., Zhang W., Ahmad A., Hao Q., Lei X., Zhang H. (2021). Structure-Guided Optimization of D-Captopril for Discovery of Potent NDM-1 Inhibitors. Bioorg. Med. Chem..

[17] Rukavishnikov A., Gee K.R., Johnson I., Corry S. (2011). Fluorogenic Cephalosporin Substrates for β-Lactamase TEM-1. Anal. Biochem..

[18] Proschak A., Kramer J., Proschak E., Wichelhaus T.A. (2018). Bacterial Zincophore [S,S]-Ethylenediamine-N,N’-Disuccinic Acid Is an Effective Inhibitor of MBLs. J. Antimicrob. Chemother..

[19] Proschak A., Martinelli G., Frank D., Rotter M.J., Brunst S., Weizel L., Burgers L.D., Fürst R., Proschak E., Sosič I. (2022). Nitroxoline and Its Derivatives Are Potent Inhibitors of Metallo-β-Lactamases. Eur. J. Med. Chem..

[20] Bohac T.J., Fang L., Giblin D.E., Wencewicz T.A. (2019). Fimsbactin and Acinetobactin Compete for the Periplasmic Siderophore Binding Protein BauB in Pathogenic Acinetobacter Baumannii. ACS Chem. Biol..

[21] Page M.G.P. (2013). Siderophore Conjugates. Ann. N. Y. Acad. Sci..

[22] Jaffe E.A., Nachman R.L., Becker C.G., Minick C.R. (1973). Culture of Human Endothelial Cells Derived from Umbilical Veins. Identification by Morphologic and Immunologic Criteria. J. Clin. Investig..

[23] Burgers L.D., Luong B., Li Y., Fabritius M.P., Michalakis S., Reichel C.A., Müller R., Fürst R. (2021). The Natural Product Vioprolide A Exerts Anti-Inflammatory Actions through Inhibition of Its Cellular Target NOP14 and Downregulation of Importin-Dependent NF-ĸB P65 Nuclear Translocation. Biomed. Pharmacother..

[24] Nicoletti I., Migliorati G., Pagliacci M.C., Grignani F., Riccardi C. (1991). A Rapid and Simple Method for Measuring Thymocyte Apoptosis by Propidium Iodide Staining and Flow Cytometry. J. Immunol. Methods.

