# Supplementary material for: Integrating Siderophore Substructures in Thiol-Based Metallo-β-Lactamase Inhibitors
Source: Molecules. 2023 Feb 20;28(4):1984. doi: 10.3390/molecules28041984 (PMC9962638; doi:10.3390/molecules28041984)

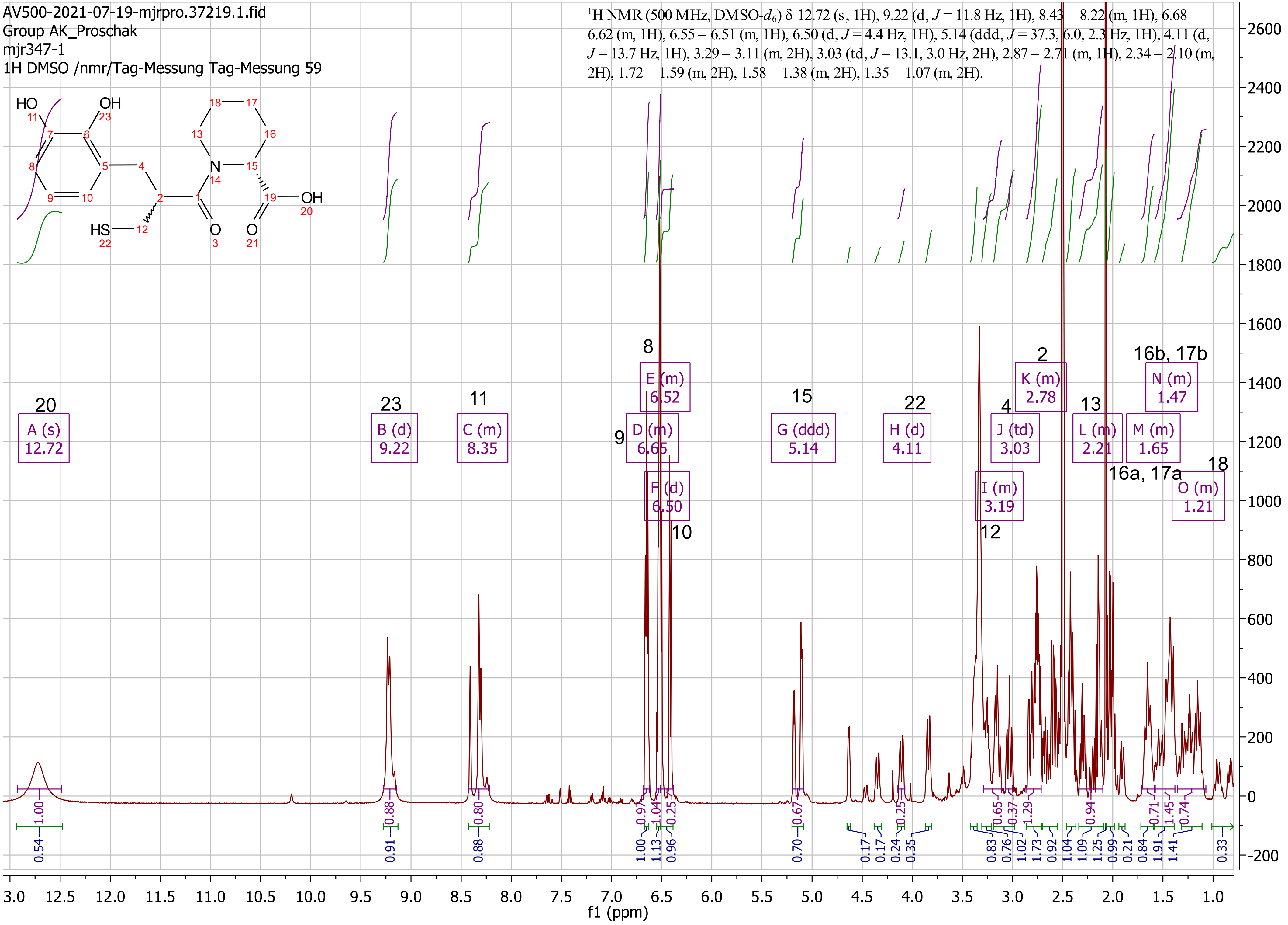
AV500-2021-07-19-mjrpro.37219.1.fid
Group AK_Proschak
mjr347-1
1H DMSO /nmr/Tag-Messung Tag-Messung 59
1H NMR (500 MHz, DMSO-d6) δ 12.72 (s, 1H), 9.22 (d, J = 11.8 Hz, 1H), 8.43 – 8.22 (m, 1H), 6.68 – 6.62 (m, 1H), 6.55 – 6.51 (m, 1H), 6.50 (d, J = 4.4 Hz, 1H), 5.14 (ddd, J = 37.3, 6.0, 2.3 Hz, 1H), 4.11 (d, J = 13.7 Hz, 1H), 3.29 – 3.11 (m, 2H), 3.03 (td, J = 13.1, 3.0 Hz, 2H), 2.87 – 2.71 (m, 1H), 2.34 – 2.10 (m, 2H), 1.72 – 1.59 (m, 2H), 1.58 – 1.38 (m, 2H), 1.35 – 1.07 (m, 2H).
A (s) 12.72
B (d) 9.22
C (m) 8.35
D (m) 6.65
E (m) 6.52
F (d) 6.50
G (ddd) 5.14
H (d) 4.11
I (m) 3.19
J (td) 3.03
K (m) 2.78
L (m) 2.21
M (m) 1.65
N (m) 1.47
O (m) 1.21
16b, 17b
16a, 17a
f1 (ppm)

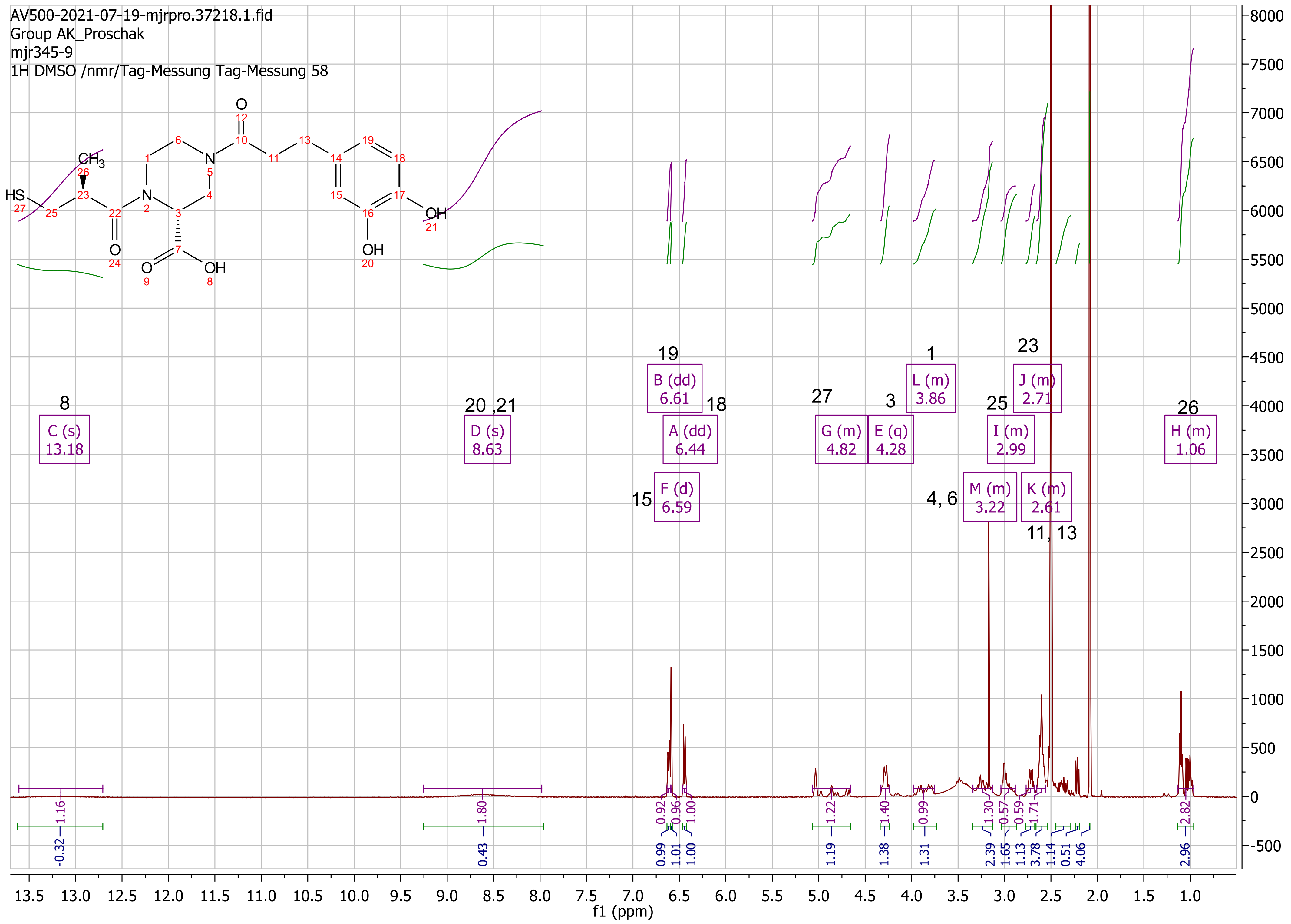
AV500-2021-07-19-mjrpro.37218.1.fid
Group AK_Proschak
mjr345-9
1H DMSO /nmr/Tag-Messung Tag-Messung 58
8
C (s)
13.18
20 ,21
D (s)
8.63
19
B (dd)
6.61
18
A (dd)
6.44
15
F (d)
6.59
27
G (m)
4.82
3
E (q)
4.28
1
L (m)
3.86
4, 6
M (m)
3.22
25
I (m)
2.99
23
J (m)
2.71
K (m)
2.61
11, 13
26
H (m)
1.06
f1 (ppm)

AV400-2021-02-11-mjrpro.39837.1.fid
Group AK_Proschak
mjr320-1
1H DMSO /nmr Tag-Messung 24

^{1}H NMR (400 MHz, DMSO-d_6) δ 7.32 (d, J = 8.1 Hz, 1H), 7.25 (d, J = 8.1 Hz, 1H), 7.13 (t, J = 8.0 Hz, 1H), 3.85 (s, 3H), 3.79 (s, 3H), 3.31 (s, 1H), 1.77 (s, 6H).

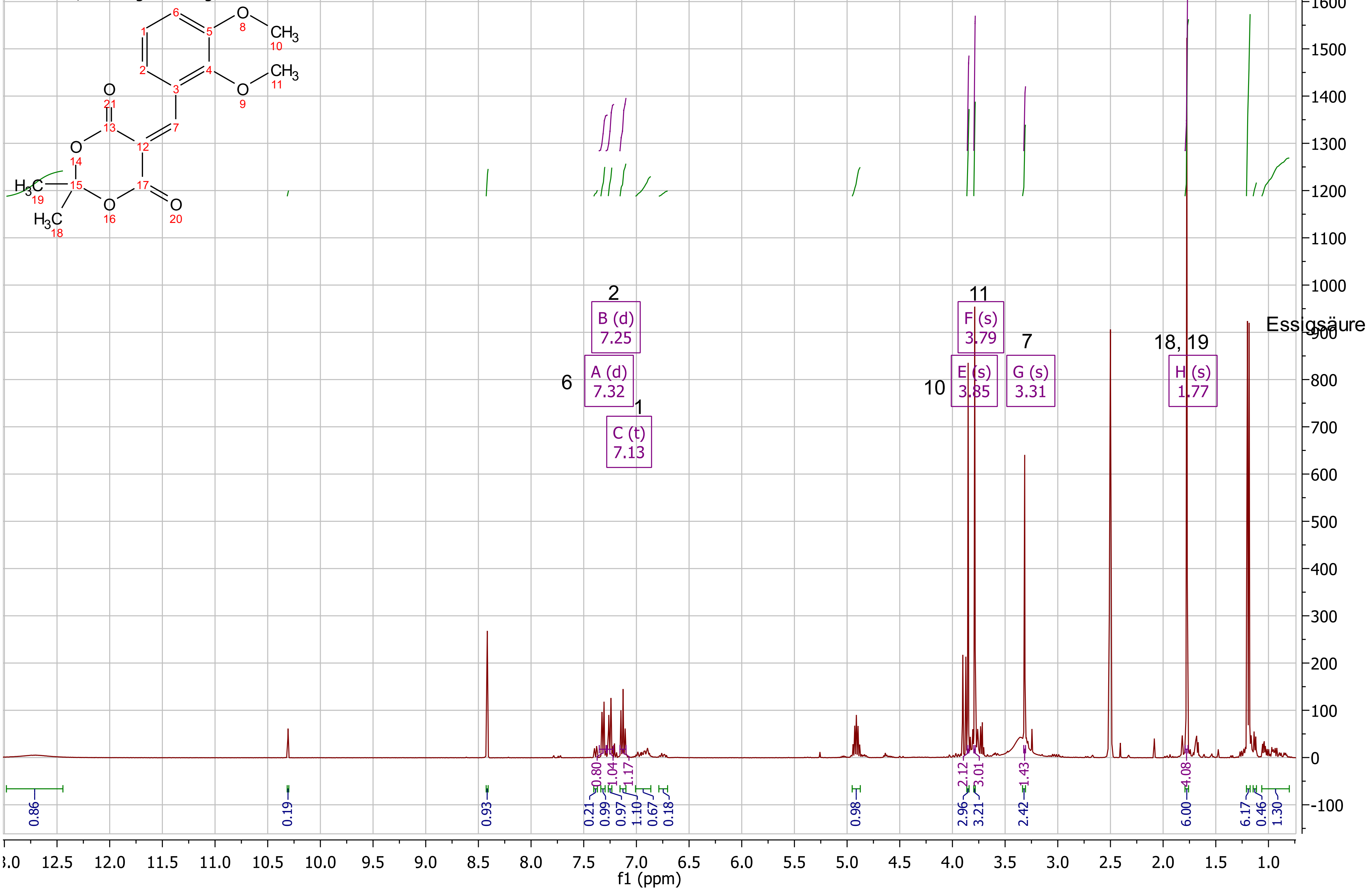

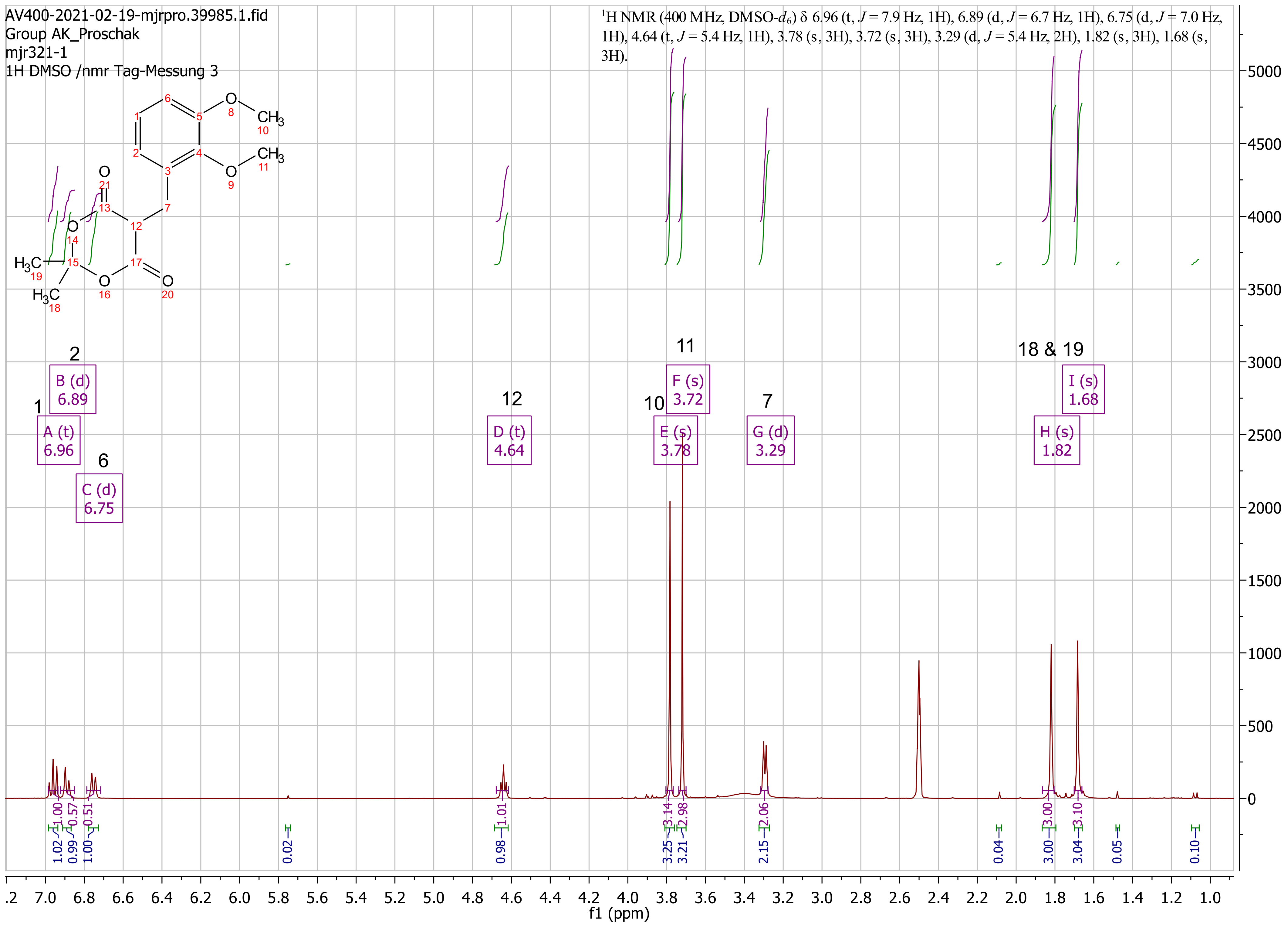
AV400-2021-02-19-mjrpro.39985.1.fid
Group AK_Proschak
mjr321-1
1H DMSO /nmr Tag-Messung 3
^{1}H NMR (400 MHz, DMSO-d_6) δ 6.96 (t, J = 7.9 Hz, 1H), 6.89 (d, J = 6.7 Hz, 1H), 6.75 (d, J = 7.0 Hz, 1H), 4.64 (t, J = 5.4 Hz, 1H), 3.78 (s, 3H), 3.72 (s, 3H), 3.29 (d, J = 5.4 Hz, 2H), 1.82 (s, 3H), 1.68 (s, 3H).
A (t) 6.96
B (d) 6.89
C (d) 6.75
D (t) 4.64
E (s) 3.78
F (s) 3.72
G (d) 3.29
H (s) 1.82
I (s) 1.68
18 & 19
f1 (ppm)

AV400-2021-02-19-mjrpro.39986.1.fid
Group AK_Proschak
mjr323-1
1H DMSO /nmr Tag-Messung 4

^{1}H NMR (400 MHz, DMSO-d_6) δ 7.01 – 6.96 (m, 1H), 6.92 (dd, J = 8.2, 1.7 Hz, 1H), 6.69 (dd, J = 7.6, 1.6 Hz, 1H), 6.14 (q, J = 1.1 Hz, 1H), 5.41 (q, J = 1.5 Hz, 1H), 3.79 (s, 3H), 3.68 (s, 3H), 3.67 (s, 3H), 3.56 (t, J = 1.2 Hz, 2H).

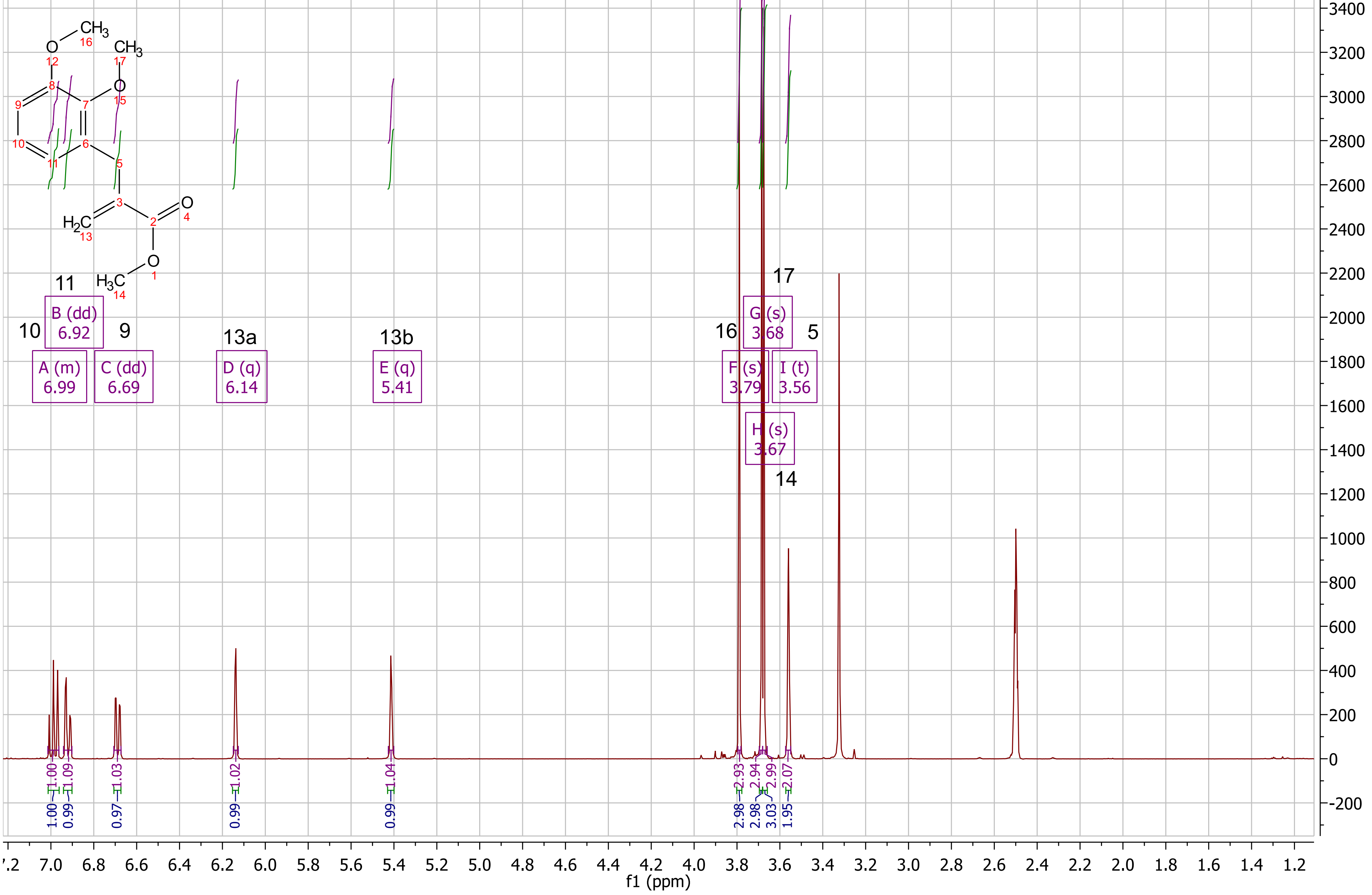

AV400-2021-02-26-mjrpro.40093.1.fid
Group AK_Proschak
mjr325-P
1H DMSO /nmr Tag-Messung 51

^{1}H NMR (400 MHz, DMSO-d_6) δ 12.49 (s, 1H), 7.01 – 6.96 (m, 1H), 6.91 (dd, J = 8.2, 1.6 Hz, 1H), 6.70 (dd, J = 7.6, 1.6 Hz, 1H), 6.13 – 6.07 (m, 1H), 5.34 (q, J = 1.6 Hz, 1H), 3.79 (s, 3H), 3.68 (s, 3H), 3.53 (s, 2H).

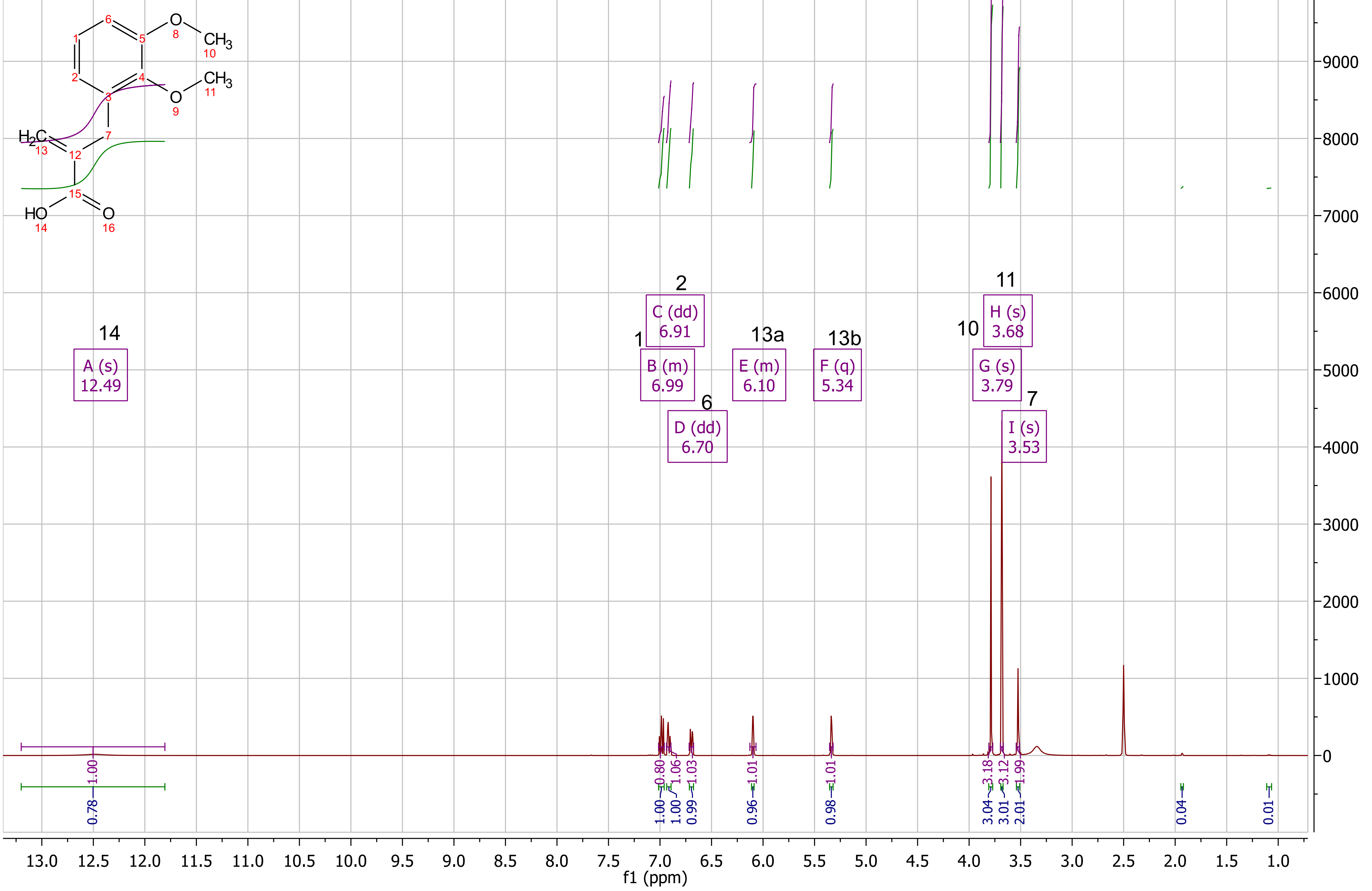

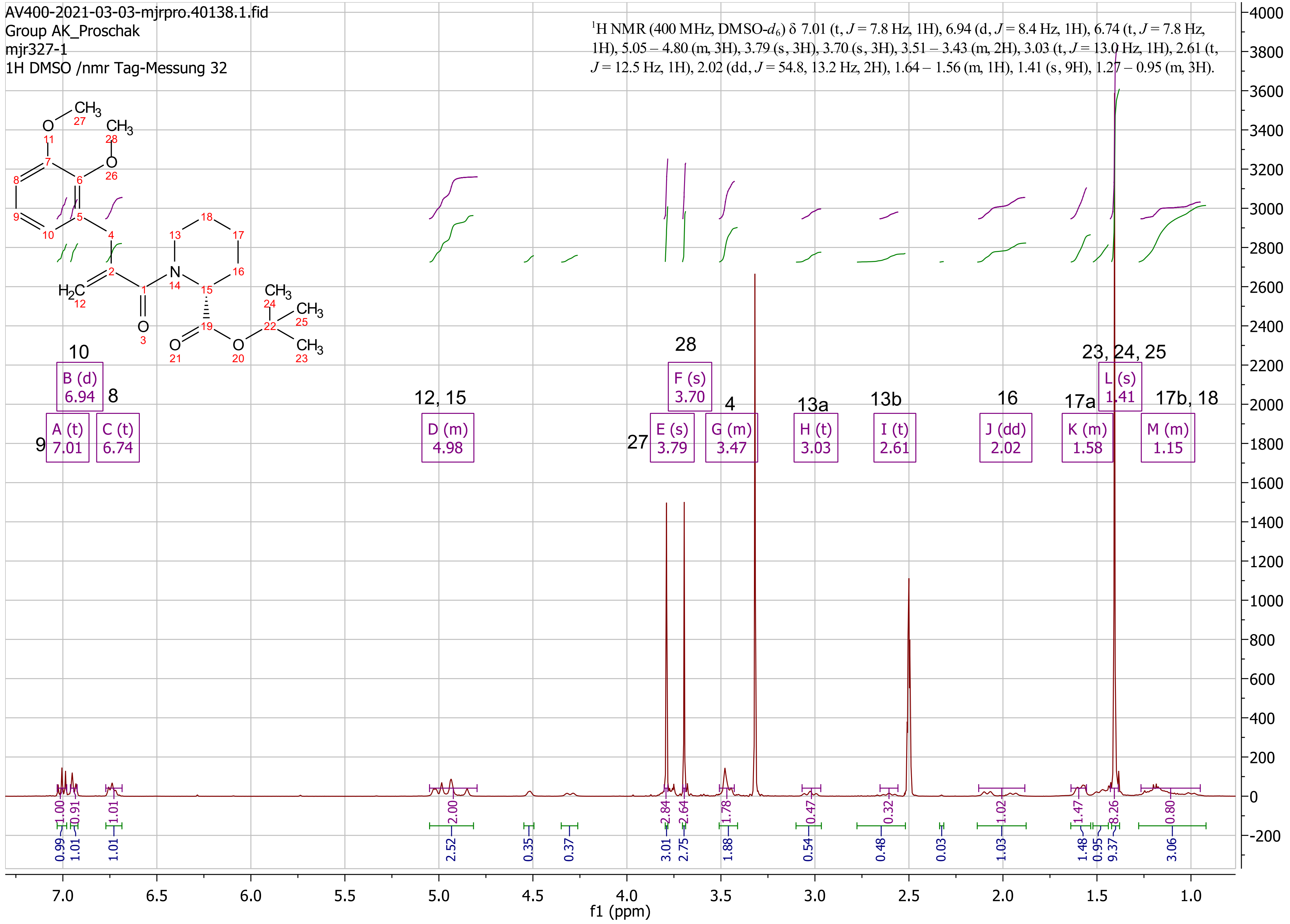
AV400-2021-03-03-mjrpro.40138.1.fid
Group AK_Proschak
mjr327-1
1H DMSO /nmr Tag-Messung 32
1H NMR (400 MHz, DMSO-d6) δ 7.01 (t, J = 7.8 Hz, 1H), 6.94 (d, J = 8.4 Hz, 1H), 6.74 (t, J = 7.8 Hz, 1H), 5.05 – 4.80 (m, 3H), 3.79 (s, 3H), 3.70 (s, 3H), 3.51 – 3.43 (m, 2H), 3.03 (t, J = 13.0 Hz, 1H), 2.61 (t, J = 12.5 Hz, 1H), 2.02 (dd, J = 54.8, 13.2 Hz, 2H), 1.64 – 1.56 (m, 1H), 1.41 (s, 9H), 1.27 – 0.95 (m, 3H).
A (t) 7.01
B (d) 6.94
C (t) 6.74
D (m) 4.98
E (s) 3.79
F (s) 3.70
G (m) 3.47
H (t) 3.03
I (t) 2.61
J (dd) 2.02
K (m) 1.58
L (s) 1.41
M (m) 1.15
f1 (ppm)

AV400-2021-05-05-mjrpro.40938.1.fid
Group AK_Proschak
mjr328-4
1H DMSO /nmr Tag-Messung 18

^{1}H NMR (400 MHz, DMSO-d_6) δ 6.98 – 6.91 (m, 2H), 6.69 (td, J = 6.7, 2.4 Hz, 1H), 5.10 – 4.98 (m, 1H), 3.79 (s, 3H), 3.74 (s, 3H), 3.27 – 3.08 (m, 2H), 3.02 – 2.87 (m, 2H), 2.74 (d, J = 7.4 Hz, 1H), 2.27 (s, 3H), 2.01 (s, 2H), 1.72 – 1.62 (m, 2H), 1.55 – 1.45 (m, 2H), 1.41 (s, 9H), 1.32 – 1.22 (m, 2H).

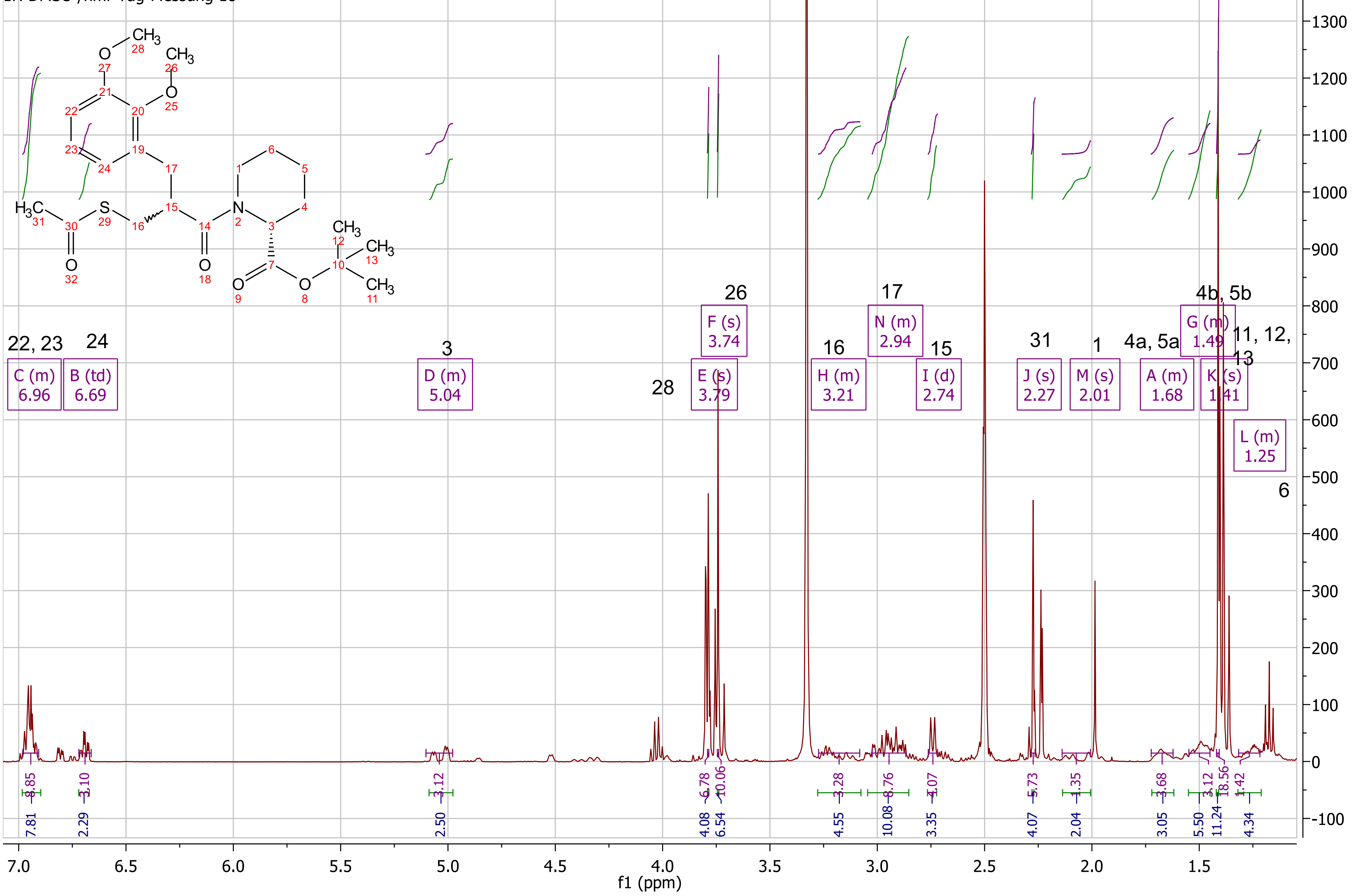

AV400-2021-09-28-mjrpro.42399.1.fid
Group AK_Proschak
mjr341
1H DMSO /nmr Tag-Messung 35

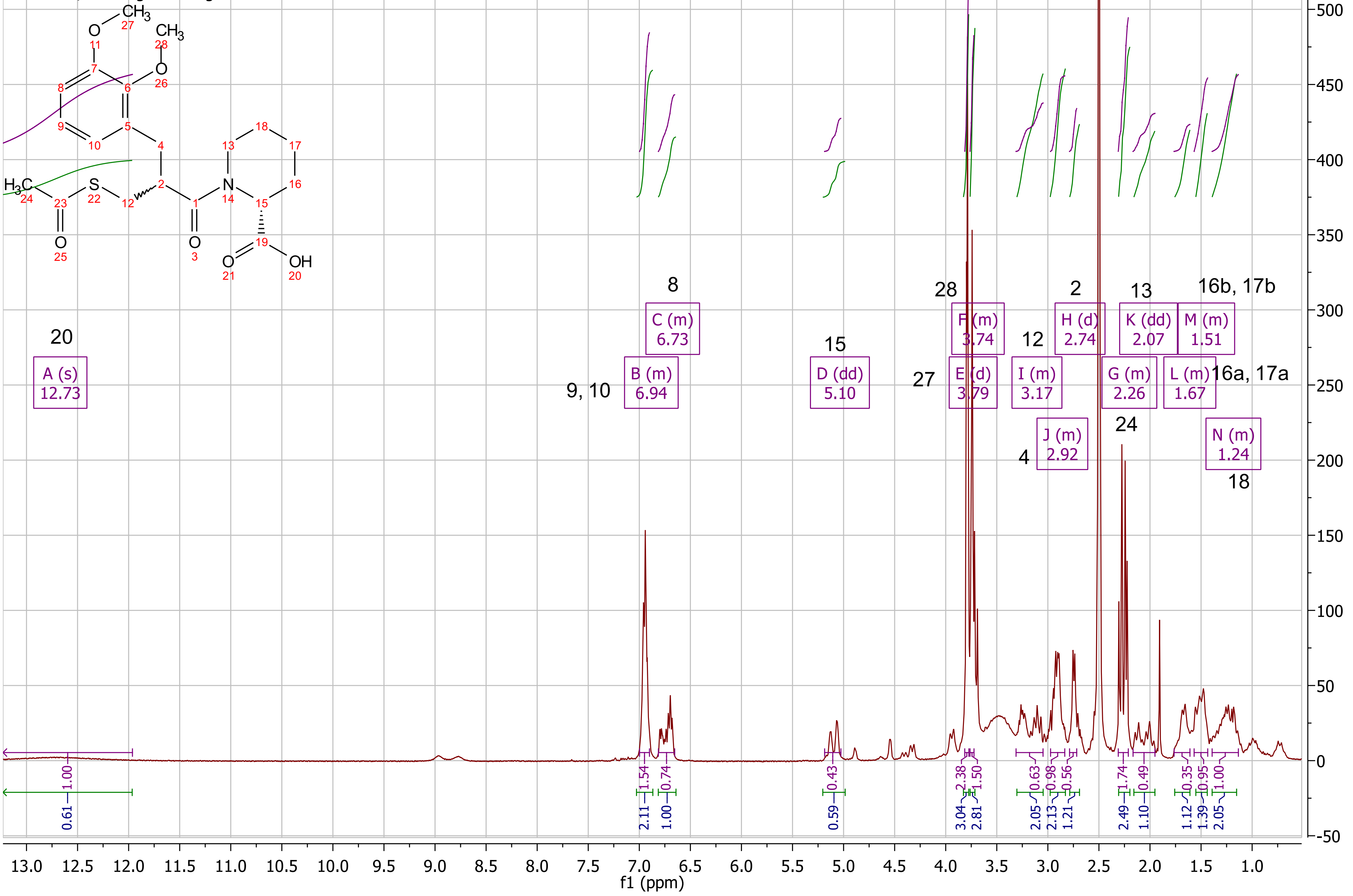

AV500-2021-07-19-mjrpro.37217.1.fid
Group AK_Proschak
mjr344-1
1H DMSO /nmr/Tag-Messung Tag-Messung 57

^{1}H NMR (500 MHz, DMSO-d_6) δ 12.80 (s, 1H), 7.00 – 6.88 (m, 2H), 6.79 – 6.68 (m, 1H), 5.14 (ddd, J = 35.7, 6.1, 2.2 Hz, 1H), 4.04 (d, J = 13.5 Hz, 1H), 3.79 (d, J = 3.2 Hz, 3H), 3.76 – 3.73 (m, 3H), 3.30 – 3.15 (m, 2H), 3.14 – 3.02 (m, 2H), 2.85 (dd, J = 13.6, 4.9 Hz, 1H), 2.12 (dd, J = 10.1, 6.9 Hz, 2H), 1.71 – 1.61 (m, 2H), 1.53 – 1.45 (m, 2H), 1.32 – 1.23 (m, 2H).

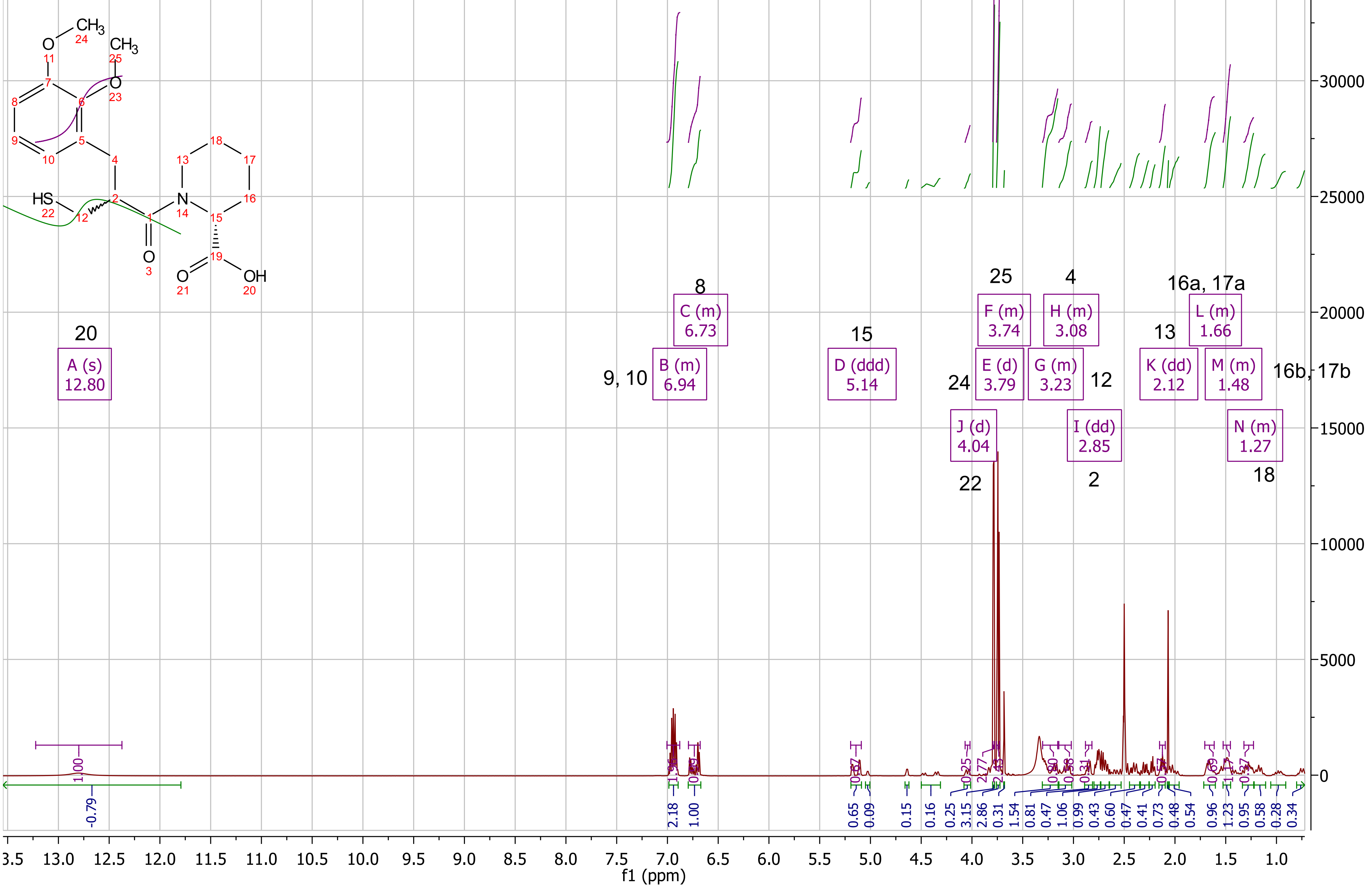

AV400-2021-02-08-mjrpro.39774.1.fid
Group AK_Proschak
mjr310-9
1H DMSO /nmr Tag-Messung 20

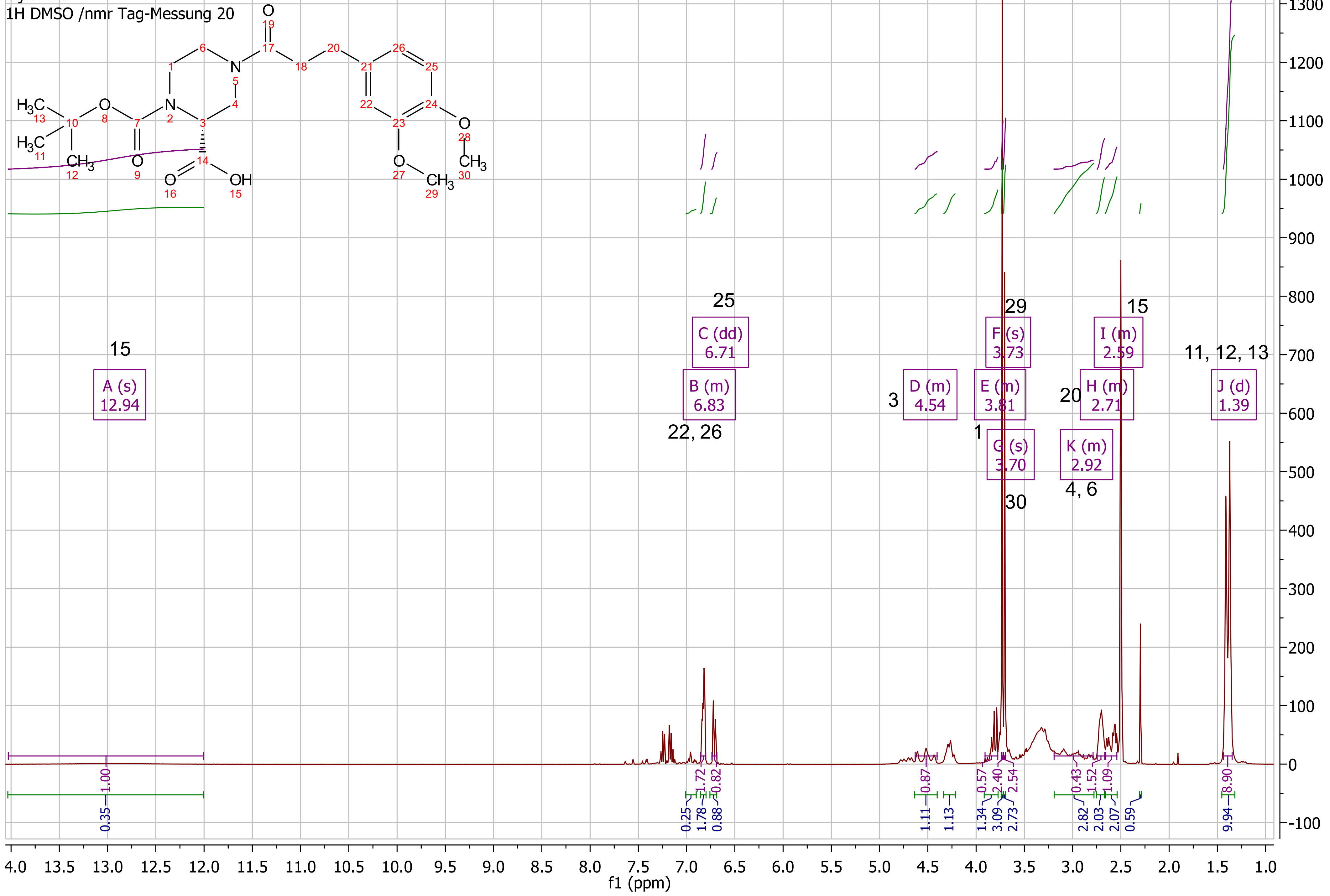

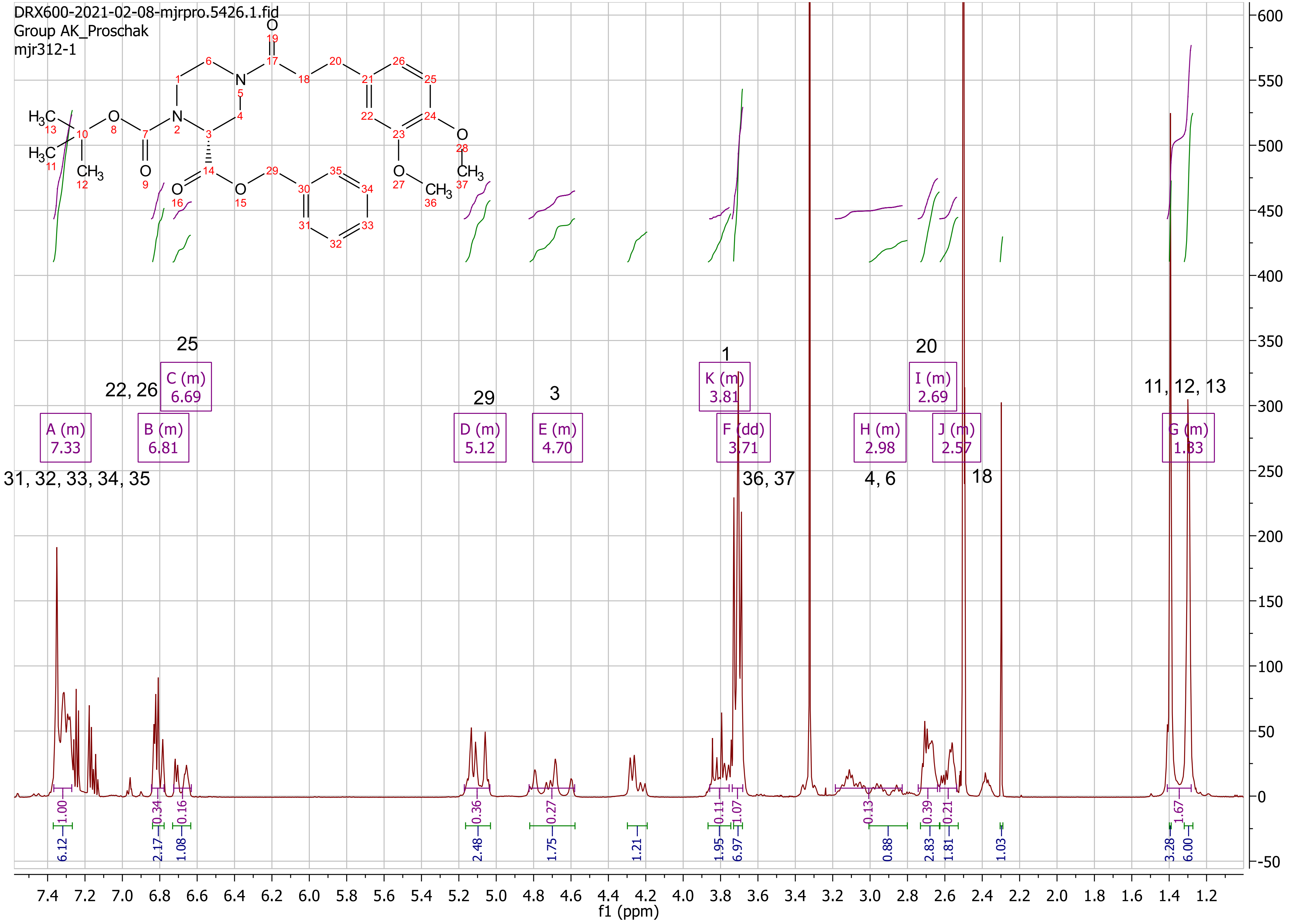

DRX600-2021-02-08-mjrpro.5426.1.fid
Group AK_Proschak
mjr312-1
A (m) 7.33
B (m) 6.81
C (m) 6.69
D (m) 5.12
E (m) 4.70
K (m) 3.81
F (dd) 3.71
H (m) 2.98
I (m) 2.69
J (m) 2.57
G (m) 1.33
31, 32, 33, 34, 35
22, 26
25
29
3
1
36, 37
4, 6
20
18
11, 12, 13
f1 (ppm)

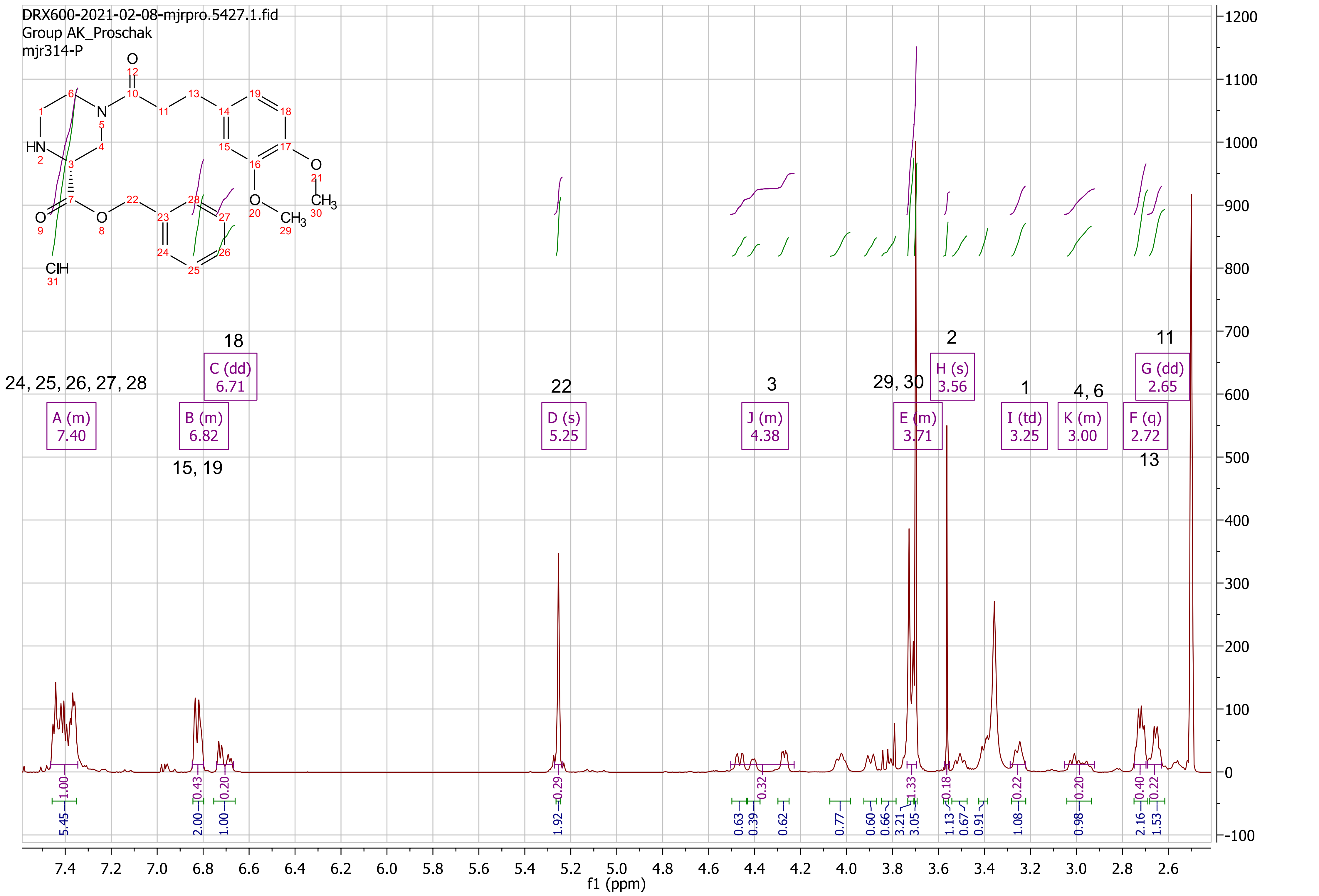

DRX600-2021-02-08-mjrpro.5427.1.fid
Group AK_Proschak
mjr314-P
24, 25, 26, 27, 28
A (m) 7.40
18
C (dd) 6.71
B (m) 6.82
15, 19
22
D (s) 5.25
3
J (m) 4.38
29, 30
E (m) 3.71
2
H (s) 3.56
1
I (td) 3.25
4, 6
K (m) 3.00
11
G (dd) 2.65
F (q) 2.72
13
f1 (ppm)

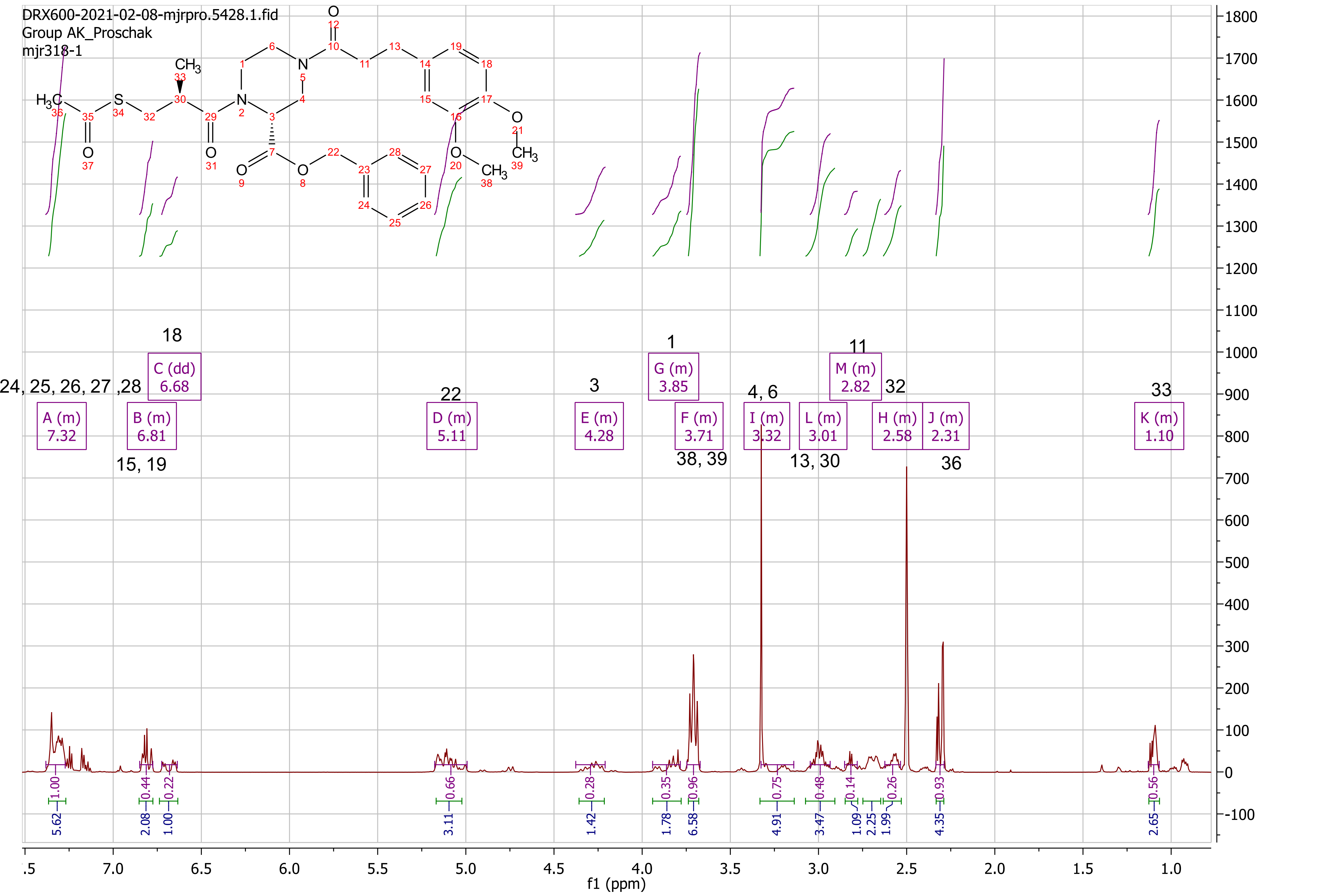

DRX600-2021-02-08-mjrpro.5428.1.fid
Group AK_Proschak
mjr318-1
A (m) 7.32
B (m) 6.81
C (dd) 6.68
D (m) 5.11
E (m) 4.28
G (m) 3.85
F (m) 3.71
I (m) 3.32
L (m) 3.01
M (m) 2.82
H (m) 2.58
J (m) 2.31
K (m) 1.10
24, 25, 26, 27 ,28
15, 19
18
22
3
1
38, 39
4, 6
13, 30
11
32
36
33
f1 (ppm)

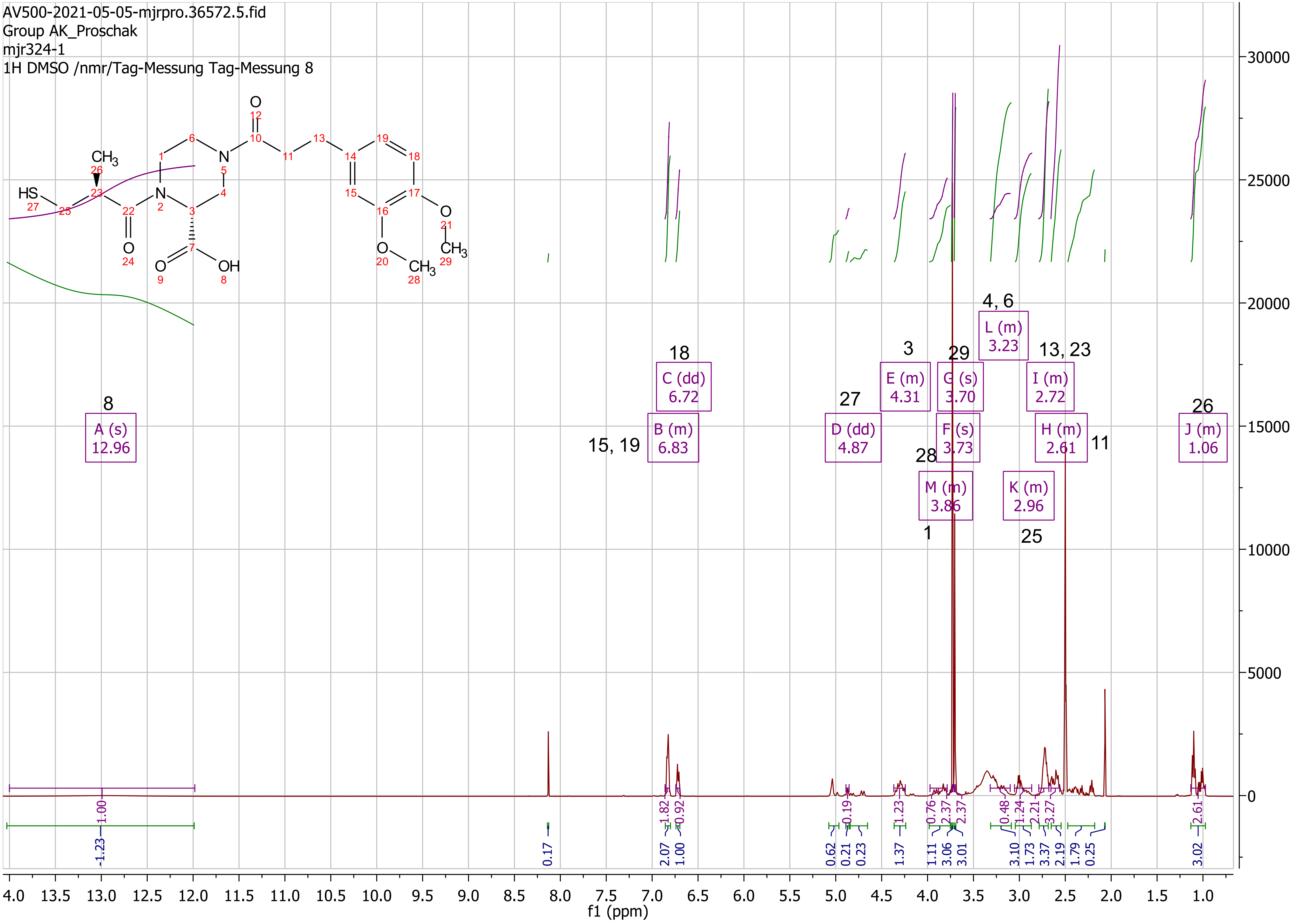

AV500-2021-05-05-mjrpro.36572.5.fid
Group AK_Proschak
mjr324-1
1H DMSO /nmr/Tag-Messung Tag-Messung 8
8
A (s) 12.96
15, 19
B (m) 6.83
18
C (dd) 6.72
27
D (dd) 4.87
3
E (m) 4.31
28
F (s) 3.73
29
G (s) 3.70
1
M (m) 3.86
4, 6
L (m) 3.23
25
K (m) 2.96
13, 23
I (m) 2.72
11
H (m) 2.61
26
J (m) 1.06
f1 (ppm)

AV400-2021-02-26-mjrpro.40094.1.fid
Group AK_Proschak
mjr326-P
1H DMSO /nmr Tag-Messung 52

^{1}H NMR (400 MHz, DMSO-d_6) δ 3.24 (dd, J = 9.7, 3.4 Hz, 1H), 2.95 (dtd, J = 12.0, 3.8, 1.3 Hz, 1H), 2.58 – 2.53 (m, 1H), 2.53 – 2.51 (m, 1H), 1.82 – 1.75 (m, 1H), 1.70 – 1.64 (m, 1H), 1.50 – 1.43 (m, 1H), 1.41 (s, 9H), 1.40 – 1.28 (m, 3H).

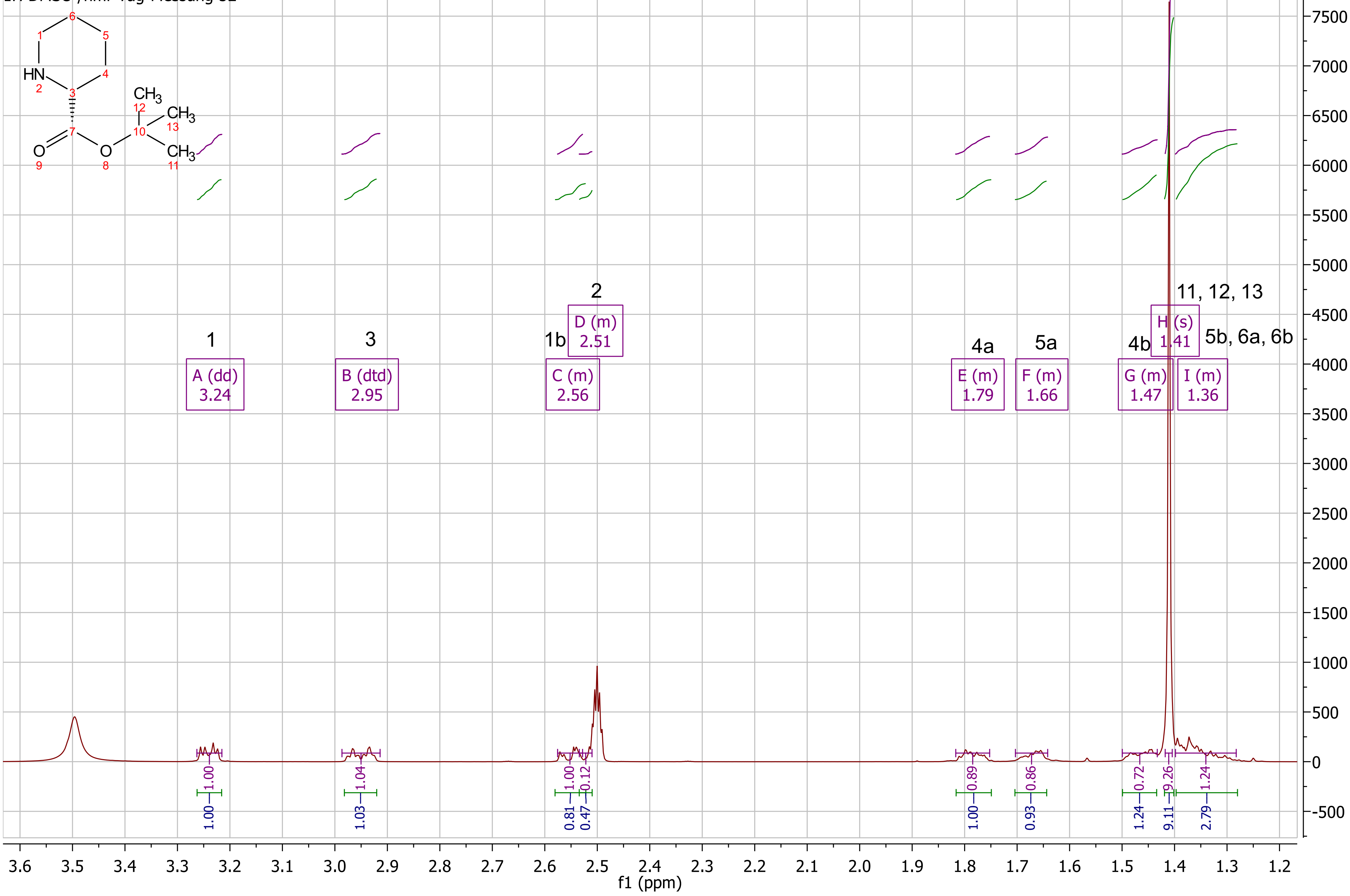

Supplement: Supplementary file 1 [file molecules-28-01984-s001.zip › molecules-2070778-supplementary.pdf]
